# Metformin impacts the differentiation of mouse bone marrow cells into macrophages affecting tumour immunity

**DOI:** 10.1016/j.heliyon.2024.e37792

**Published:** 2024-09-11

**Authors:** Andrea Scafidi, Frida Lind-Holm Mogensen, Eleonora Campus, Alexandros Pailas, Katrin Neumann, Nathalie Legrave, François Bernardin, Sandro L. Pereira, Paul M.A. Antony, Nathalie Nicot, Michel Mittelbronn, Anne Grünewald, Petr V. Nazarov, Aurélie Poli, Eric Van Dyck, Alessandro Michelucci

**Affiliations:** a Neuro-Immunology Group, Department of Cancer Research, Luxembourg Institute of Health, L-1210 Luxembourg, Luxembourg; b Faculty of Science, Technology and Medicine, University of Luxembourg, L-4365 Esch-sur-Alzette, Luxembourg; c DNA Repair and Chemoresistance, Department of Cancer Research, Luxembourg Institute of Health, L-1210 Luxembourg, Luxembourg; d Metabolomics Platform, Department of Cancer Research, Luxembourg Institute of Health, L-1445 Strassen, Luxembourg; e Molecular and Functional Neurobiology Group, Luxembourg Centre for Systems Biomedicine, University of Luxembourg, L-4362 Esch-sur-Alzette, Luxembourg; f Bioimaging Platform, Luxembourg Centre for Systems Biomedicine, University of Luxembourg, L-4362 Esch-sur-Alzette, Luxembourg; g LuxGen Genome Center, Luxembourg Institute of Health & Laboratoire National de Santé, L-3555 Dudelange, Luxembourg; h Department of Life Sciences and Medicine, Faculty of Science, Technology and Medicine, University of Luxembourg, L-4367 Belvaux, Luxembourg; i Luxembourg Centre for Systems Biomedicine, University of Luxembourg, L-4362 Esch-sur-Alzette, Luxembourg; j Department of Cancer Research, Luxembourg Institute of Health, L-1210 Luxembourg, Luxembourg; k Luxembourg Center of Neuropathology, Laboratoire National de Santé, L-3555 Dudelange, Luxembourg; l National Center of Pathology, Laboratoire National de Santé, L-3555 Dudelange, Luxembourg; m Bioinformatics and AI unit, Department of Medical Informatics, Luxembourg Institute of Health, L-1445 Strassen, Luxembourg; n Multiomics Data Science Group, Department of Cancer Research, Luxembourg Institute of Health, L-1445 Strassen, Luxembourg

**Keywords:** **Metformin**, **Macrophages**, **Phagocytosis**, **Anti-tumour**, **HIF1α**, **Glycolysis**

## Abstract

**Background:**

Epidemiological studies suggest that metformin reduces the risk of developing several types of cancer, including gliomas, and improves the overall survival in cancer patients. Nevertheless, while the effect of metformin on cancer cells has been extensively studied, its impact on other components of the tumour microenvironment, such as macrophages, is less understood.

**Results:**

Metformin-treated mouse bone marrow cells differentiate into spindle-shaped macrophages exhibiting increased phagocytic activity and tumour cell cytotoxicity coupled with modulated expression of co-stimulatory molecules displaying reduced sensitivity to inflammatory cues compared with untreated cells. Transcriptional analyses of metformin-treated mouse bone marrow-derived macrophages show decreased expression levels of pro-tumour genes, including *Tgfbi* and *Il1β*, related to enhanced mTOR/HIF1α signalling and metabolic rewiring towards glycolysis.

**Significance:**

Our study provides novel insights into the immunomodulatory properties of metformin in macrophages and its potential application in preventing tumour onset and in cancer immunotherapy.

## 1 Introduction

Metformin (1,1-dimethylbiguanide hydrochloride) is the primary oral treatment for patients with type 2 diabetes for its inhibitory activity toward hepatic gluconeogenesis [1]. Besides its anti-hyperglycaemic effect, metformin reduces the risk of development of several cancer types and improves the overall survival in both tumour murine models and oncologic patients [2]. In fact, metformin has been found to inhibit the proliferation of colon, breast, prostate and pancreatic cancer cells, as well as leukaemia, melanoma, lung cancer, endometrial carcinoma and glioma cells [3]. The *in vitro* effects of metformin have been extensively studied in cancer cells where, for example, it inhibits the tumorigenic activity of the mammalian target of rapamycin (mTOR) [4], as well as in the immune components of the tumour microenvironment (TME), where it reduces the amounts of myeloid-derived suppressor cells, enhances the pro-inflammatory features of tumour-associated macrophages [5], downregulates regulatory T cell functions, enhances NK cell anti-tumour activity [6] and decreases the expression levels of immune checkpoints [7]. Nonetheless, the effect of metformin on the differentiation of bone marrow-derived macrophages, which play critical roles in tumour immunity [8] and therefore may contribute to decrease the risk of cancer development and progression, remains elusive.

Although specific stimuli can polarize macrophages towards “classical” or “alternative” activation states characterized, respectively, by pro-inflammatory or wound healing activities [9], macrophages in the TME display polarization states with aspects of both phenotypes [10], as they respond to a plethora of stimuli depending on the specific niches, such as hypoxic regions, which simultaneously induce the expression of pro-inflammatory cytokines, such as tumour necrosis factor-alpha (TNFα) and interleukin 1 beta (IL1β), and wound-healing factors, including vascular endothelial growth factor A (VEGFA), tumour growth factor beta (TGFβ) and TGFβ induced (TGFBI) [[11], [12], [13]]. In hypoxic areas, macrophages are reprogrammed by the activity of hypoxia inducible factor 1 alpha (HIF1α), which in normoxic conditions undergoes proteasomal degradation, while under hypoxia it acts as a transcriptional regulator [11,12,14].

Here, we elucidate the effect of metformin *in vitro* on the differentiation of mouse bone marrow cells towards macrophages, which represent well-established mediators that precede the onset of cancer and shape its evolution [15]. The reported impact of metformin on cancer risk reduction and cancer survival rate improvement suggests that macrophages differentiated in the presence of metformin acquire a specific anti-tumour phenotype, both at the molecular and functional level. In line with this concept, we show that metformin-treated bone marrow cells under differentiating conditions segregate into spindle-shaped macrophages with enhanced phagocytic activity and anti-glioma cell cytotoxicity coupled with modulated expression of co-stimulatory molecules when compared with untreated counterparts. Furthermore, a distinct immune profile, mainly characterized by reduced responsiveness towards inflammatory cues, enhanced mTOR/HIF1α signalling, and glycolysis-based metabolism distinguishes their phenotype. Taken together, the combination of these macrophage characteristics may contribute to the reduced tumour incidence and delayed tumour growth observed in metformin-treated individuals.

## 2 Results

### 2.1 Metformin-treated bone marrow cells differentiate into macrophages with distinct functional marks

**Fig. 1:**
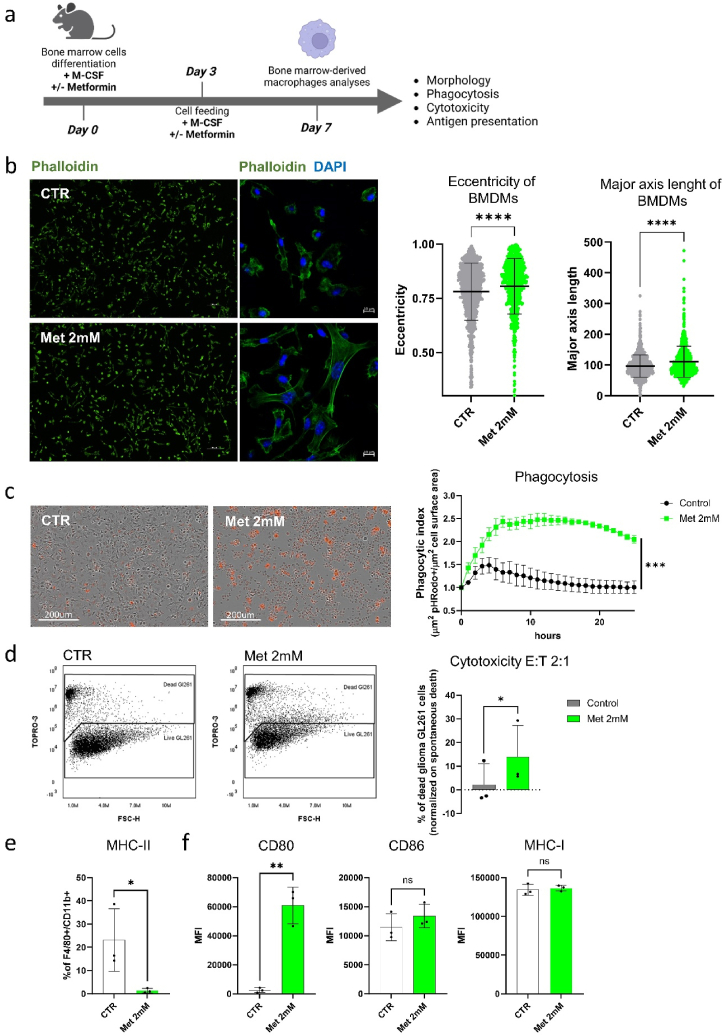
Metformin-treated bone marrow cells differentiate into macrophages displaying distinct functional properties compared with untreated cells. **(A)** Schematic of differentiation and treatment protocol of bone marrow cells towards macrophages. Bone marrow cells were collected from C57BL/6N mouse femurs and tibia subsequently cultured for 7 days in the presence of M-CSF (20 ng/mL) with or without metformin (2 mM). **(B)** Left: representative pictures of phalloidin-stained untreated or metformin-treated BMDMs. Scale bar: 100 μm. Right: quantification of BMDM morphological parameters eccentricity (value of 0 represents a circle, while value of 1 represents an ellipse or an elongated shape) and major axis length. Two-tailed Mann-Whitney *U* test. Graphs show mean ± SD. **** = p < 0.0005 (n = 3 biological replicates with ≥300 cells per image/condition). **(C)** Left: representative pictures of untreated or metformin-treated pHRodo^+^ BMDMs after 25h of incubation with fluorescent beads. Scale bar: 200 μm. Right: normalised phagocytic index quantified using Incucyte™ analysis software. 2-way ANOVA. Graph shows phagocytic index ± SD. **** = p < 0.0005 (n = 3 biological replicates). **(D)** Left: representative dot plots of GL261 glioma cells after co-culture with BMDMs untreated or treated with metformin. Right: percentage of dead GL261 glioma cells. Paired *t*-test. Graph shows mean percentage ± SD. * = p < 0.05 (n = 3 biological replicates). **(E)** Graph shows mean percentages of MHC class II^+^ BMDMs ± SD. Unpaired *t*-test. * = p < 0.05 (n = 3 biological replicates). **(F)** Graphs show geometrical mean fluorescence intensity (MFI) ± SD of CD80, CD86, and MHC class I. Unpaired *t*-test. Ns = p > 0.05, ** = p < 0.01 (n = 3 biological replicates).

To analyse the immunological functions of macrophages differentiated in the presence of metformin, we collected bone marrow cells from the femurs and tibias of C57BL/6N female mice and seeded them under differentiating conditions in the presence of M-CSF (20 ng/mL) with or without metformin (2 mM) [16]. We then analysed their resulting morphological and immunological characteristics (Fig. 1A).

First, using flow cytometry we observed that although metformin did not impair the viability of macrophages after 7 days of differentiation, it led to increased percentages of CD11b^+^F4/80^+^ differentiated macrophages compared to control conditions, indicating that metformin either decreased the survival of undifferentiated cells or it enhanced their differentiation (Fig. S1A). At the morphological level, bone marrow-derived macrophages (BMDMs) differentiated in the presence of metformin showed longer protrusions and a spindle-shaped morphology (Fig. 1B). To assess if these morphological changes were reflected at the functional level, we compared the phagocytic capacity of metformin-treated BMDMs versus untreated cells using *E. coli*-covered pHRodo beads. In both cases, the phagocytic activity reached the plateau between 3 and 5 h. However, while only a fraction of untreated BMDMs showed phagocytic activity along the studied time points (e.g. 1.01 ± 0.08 phagocytic index normalised to initial state), metformin-treated BMDMs displayed an increased phagocytic response already within the first hours of incubation with a significant higher phagocytic index across all the analysed time points (e.g. 2.05 ± 0.05 phagocytic index normalised to initial state) (Fig. 1C). Next, to investigate the ability of metformin-treated macrophages to actively promote tumour cell death, we co-cultured fully differentiated macrophages with CFSE-labelled murine glioma GL261 cells at effector-target ratio 1:1 and 2:1 to evaluate viability of tumour cells after 24 h. No differences in GL261 cell survival were observed between metformin-treated and untreated BMDMs at a 1:1 ratio (Fig. S1B). However, when glioma cells were challenged with higher amounts of macrophages (ratio 2:1), metformin-treated BMDMs led to a significant glioma cell death when compared with untreated macrophages, which displayed low cytotoxicity or even improved tumour cell survival (percentage of dead glioma GL261 cells: 13.88 ± 7.6 % when co-cultured with metformin-treated BMDMs and 2.1 ± 5.1 % with untreated BMDMs) (Fig. 1D). Lastly, we investigated the antigen presenting cell properties of metformin-treated BMDMs by analysing the expression levels of major histocompatibility complex (MHC) class I (H-2K^b^/H-2D^b^) and class II (H-2^b^) molecules, as well as the co-stimulatory molecules CD80 and CD86 using multicolor flow cytometry. Metformin-treated BMDMs displayed a reduction of MHC class II^+^ cell fraction (Fig. 1E/S1C), increased expression levels of CD80, while CD86 and MHC class I molecules were not modified when compared with untreated cells (Fig. 1F/S1D).

Collectively, these results show that metformin-treated bone marrow cells differentiate into spindle-shaped macrophages displaying increased phagocytic activity and glioma cell cytotoxicity together with modulated expression of MHC class I and CD80 molecules, suggesting that these cells acquire a distinct immunological phenotype when compared with corresponding untreated macrophages.

### 2.2 Metformin reduces bone marrow-derived macrophage sensitivity to pro- and anti-inflammatory cues

**Fig. 2:**
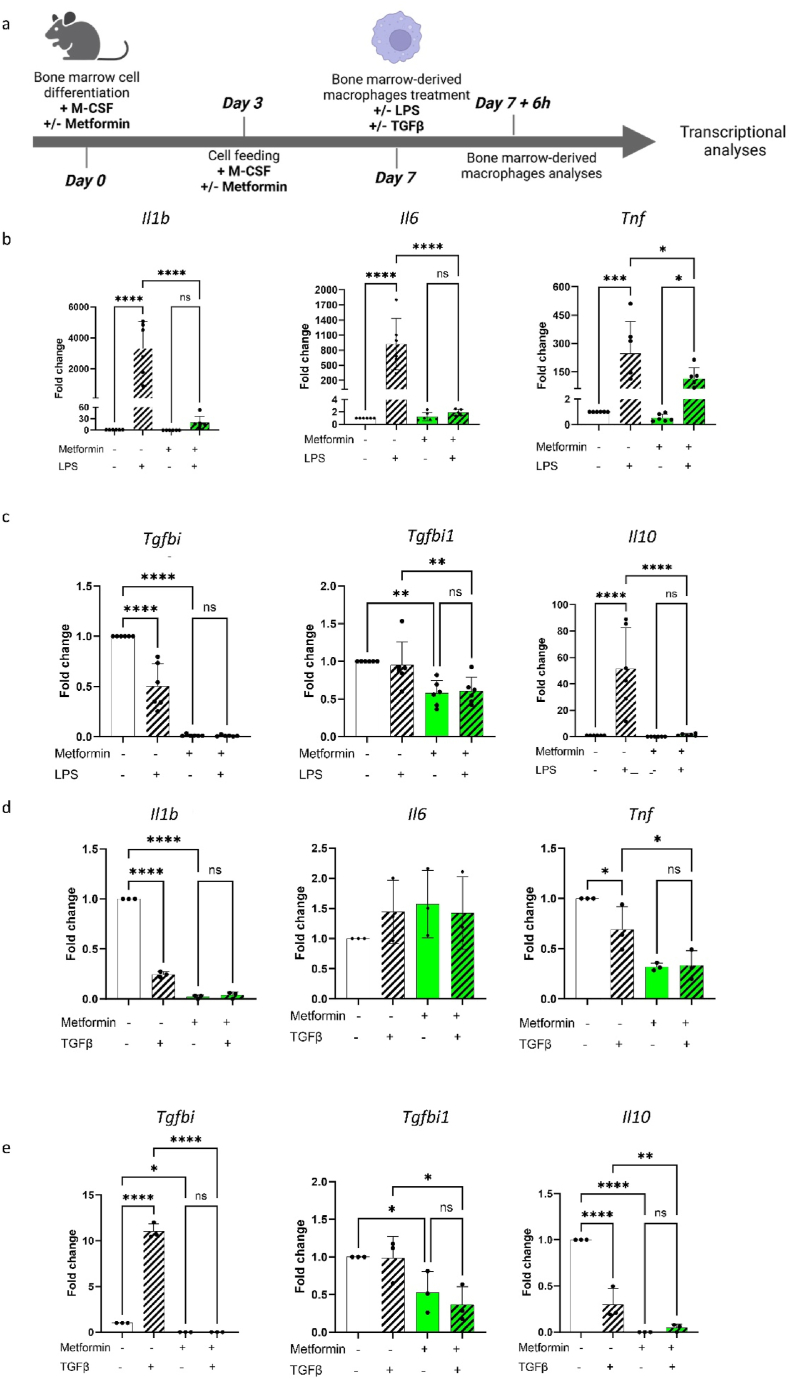
Metformin impairs bone marrow-derived macrophage polarization. **(A)** Schematic protocol of differentiation and treatment of bone marrow cells towards their differentiation into macrophages and polarizing treatment. Bone marrow cells were collected from C57BL/6N mouse femurs and tibia subsequently cultured for 7 days in the presence of M-CSF (20 ng/mL) with or without metformin (2 mM). Following differentiation, BMDMs were treated for 6h with either LPS or TGFβ for subsequent gene expression analyses. **(B**–**C)** Relative quantification of mRNA expression of **(B)** pro-inflammatory cytokines *Il1b*, *Ccl2*, *Tnf* and **(C)** anti-inflammatory genes *Tgfbi*, *Tgfb1*, *Il10* after metformin and/or LPS treatment. One-way ANOVA. Graphs show fold change compared to untreated BMDMs ± SD. Ns = p > 0.05, * = p < 0.05, ** = p < 0.01, *** = p < 0.001, **** = p < 0.0005 (n = 6 biological replicates). **(D**–**E)** Relative quantification of mRNA expression of **(D)** pro-inflammatory cytokines *Il1b*, *Ccl2*, *Tnf* and **(E)** anti-inflammatory genes *Tgfbi*, *Tgfb1* and *Il10* after metformin and/or TGFβ treatment. One-way ANOVA. Graphs show fold change compared to untreated BMDMs ± SD. Ns = p > 0.05, * = p < 0.05, ** = p < 0.01, *** = p < 0.001, **** = p < 0.0005 (n = 3 biological replicates).

Next, we sought to elucidate the responsiveness of macrophages differentiated from metformin-treated bone marrow cells to a pro- or anti-inflammatory stimulus. To this aim, we respectively treated differentiated macrophages that have been previously treated with metformin or untreated macrophages either with LPS or TGFβ for 6 h (Fig. 2A). When activated with LPS (10 ng/mL), metformin-treated BMDMs showed a strong dampening of the pro-inflammatory response compared with metformin-untreated BMDMs, as exemplified by reduced expression levels of *Il1b*, *Il6* and *Tnf* (Fig. 2B), together with a dampened anti-inflammatory response, characterized by the expression of immune-modulating cytokine genes, such as *Tgfbi*, *Tgfb1* and *Il10*, both at basal level and upon stimulation with LPS (Fig. 2C). On the other hand, after treatment with TGFβ (10 ng/mL), metformin-treated BMDMs displayed a stronger downregulation of *Il1β* and *Tnf* compared with metformin-untreated BMDMs (Fig. 2D), together with decreased expression levels of *Tgfbi* and *Il10* (Fig. 2E).

Taken together, these results show that metformin-treated BMDMs display a reduced capacity of polarizing towards pro- or anti-inflammatory states when exposed to corresponding inflammatory cues.

### 2.3 Metformin reduces expression levels of macrophage pro-tumour genes and increases mTOR/HIF1α pathway

**Fig. 3:**
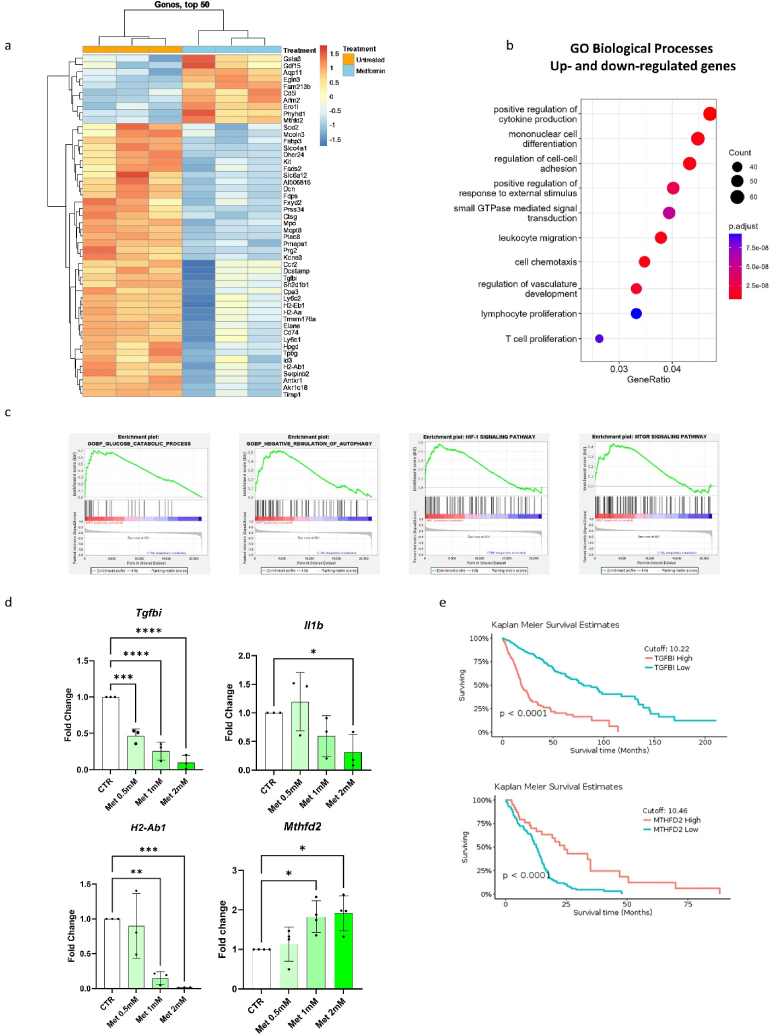
Metformin induces specific bone marrow-derived macrophage phenotypical and transcriptional adaptation related to autophagy and HIF1α programs. **(A)** Heatmap showing top 50 differentially expressed genes between metformin-treated and untreated bone marrow-derived macrophages (p value < 0.05; |logFC| ≥ 0.5). Color bar shows z-scores of expression. **(B)** Dot plot showing top 10 modulated gene ontology (GO) biological processes related to up- and down-regulated genes between metformin-treated and untreated bone marrow-derived macrophages (circles denote number of genes within each specific GO term and color bar shows adjusted p value). **(C)** Gene set enrichment analysis (GSEA) of representative gene ontology biological processes (enrichment plot 1 and 2) and KEGG pathway (enrichment plot 3 and 4) upregulated in metformin-treated bone marrow-derived macrophages compared to control. (**D)** Relative quantification of mRNA expression of *Tgfbi*, *Il1b, H2-Ab1* and *Mthfd2* comparing macrophages treated with different concentrations of metformin (0.5, 1 and 2 mM) with untreated cells. One-way ANOVA. Graphs show fold change compared to untreated BMDMs ± SD. * = p < 0.05, ** = p < 0.01, *** = p < 0.001, **** = p < 0.0005 (n = 3/4 biological replicates). **(E)** Kaplan Meier curves showing survival time of glioma patients stratified based on *TGFBI* and *MTHFD2* mRNA expression in the tumour tissue. (For interpretation of the references to color in this figure legend, the reader is referred to the Web version of this article.)

Since BMDMs differentiated in the presence of metformin displayed anti-tumour functions and were less prone to be polarized by inflammatory stimuli, we further explored the impact of this drug on their transcriptomic profile using a microarray approach. Among the 1′470 detected differentially expressed genes (DEGs) (p value < 0.05; |logFC| ≥ 0.5), 841 genes were up- and 629 genes were down-regulated when comparing metformin-treated with untreated cells (Fig. S2A, Table S1). We visualized the expression pattern of the top 50 DEGs in a heat map (Fig. 3A). To identify the biological processes that were modulated by metformin treatment, we performed gene ontology enrichment analysis [17]. BMDMs derived from metformin-treated cells exhibited modulation of biological processes associated with cytokine production, cell-cell adhesion, angiogenesis, and T cell proliferation, in line with the observed impact of metformin on macrophage functions (Fig. 3B–Table S2).

Furthermore, we carried out gene set enrichment analysis (GSEA) [18,19] taking into account gene ontology biological processes and Kyoto Encyclopedia of Genes and Genome (KEGG) pathways to infer the most pronounced up- and down-regulated transcriptional programs in the comparison of metformin-treated versus control BMDMs. We identified enrichment of genes related to the HIF1α and mTOR pathways as well as genes involved in metabolic processes, including glucose catabolism, glycolysis, and negative regulation of autophagy (Fig. 3C, Fig. S2B, Table S3).

To investigate whether the dosage of metformin influenced the identified transcriptional reprogramming, we cultured bone marrow cells with three different concentrations of metformin (0.5, 1 and 2 mM). Then, we evaluated by qPCR the main modulated genes that we identified in our transcriptomic analyses, which are related to essential macrophage functions and metabolism. The analysis of the expression levels of *Tgfbi*, *Il1b, H2-Ab1, Timp1*, *H2-Aa*, *Ly6c1,* and *Cd74* showed a dose-dependent decrease, while *Mthfd2* was increased, when compared with corresponding untreated cells (Fig. 3D and Fig. S2D). Moreover, we took advantage of the GlioVis portal [20] to examine the correlation between levels of expression of metformin-modulated genes and survival of glioma patients, according to TCGA database. High expression levels of genes downregulated by metformin in macrophages, such as *TGFBI*, *IL1B*, *CD74* and *TIMP1*, correlated with poor overall survival in glioma patients, as did low expression of the upregulated gene *MTHFD2* (Fig. 3E and Fig. S2E), thus suggesting that the beneficial effects on the prognosis of metformin-treated glioma patients may be related to its action on macrophages.

Lastly, a comparison of metformin-treated BMDM transcriptomic profile with prototypical M1-like and M2-like macrophage signatures [21], showed a prominent downregulation of M2 signatures (normalised enrichment score = −2.46, FDR<0.0001), whereas displayed a slight enrichment of the M1 signature (normalised enrichment score = 1.36, FDR<0.05) (Fig. S2C).

Taken together, these results indicate that metformin-treated BMDMs display a distinct transcriptional pattern compared to untreated BMDMs, which is related to macrophage functions, such as antigen presentation, cytokine production and metabolic rewiring through HIF1α and mTOR signalling pathways.

### 2.4 Metformin-treated bone marrow cells differentiate into macrophages engaging a mTOR/HIF1α program

**Fig. 4:**
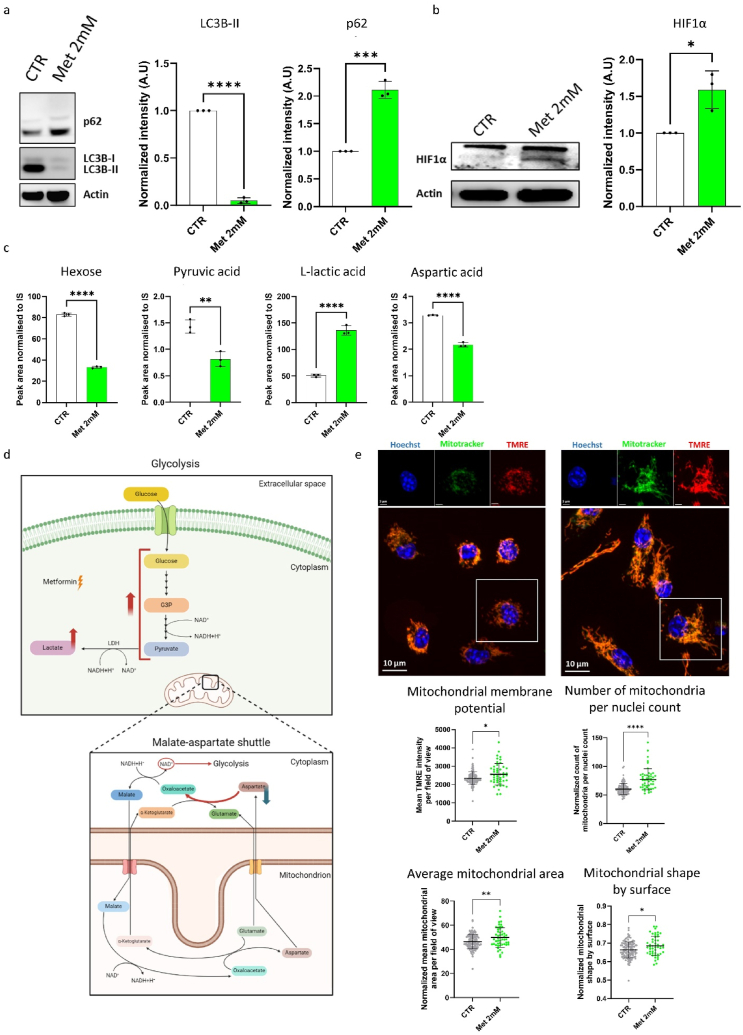
Metformin induces HIF1α activation and autophagy inhibition in bone marrow-derived macrophages. **(A)** Western blot gel showing the corresponding bands for LC3B-I, LC3B-II, p62 and β-ACTIN proteins and quantification. Unpaired *t*-test. Graphs show mean relative expression ± SD. *** = p < 0.001, **** = p < 0**.**0005 (n = 3 biological replicates). **(B)** Western blot gel showing the corresponding bands for HIF1α and β-ACTIN proteins and quantification of HIF1α normalised to β-ACTIN expression. Unpaired *t*-test. Graphs show mean relative expression ± SD. * = p < 0**.**05 (n = 3 biological replicates). **(C)** Quantification of hexoses, pyruvic acid, L-lactic acid and aspartic acid in the supernatant of untreated and metformin-treated BMDMs. Unpaired *t*-test. Graphs show mean relative quantification ± SD. ** = p < 0.01, **** = p < 0.0001 (n = 3 biological replicates). **(D)** Scheme depicting the metabolic rewiring induced in bone marrow-derived macrophages treated 7 days with metformin. Red arrows indicate induced metabolic pathway or metabolite, while the blue arrow depicts the inhibited metabolite. **(E)** Upper panel: representative pictures of untreated (left) and metformin-treated BMDMs (right) stained with Hoechst, MitoTracker green FM, and TMRE. Scale bar: 3 μm (single colors) or 10 μm (merged colors). Analysis of mitochondrial membrane potential (MMP) using TMRE tracer per field of view (dots) in metformin-treated BMDMs compared to untreated cells. The number and morphology of mitochondria were assessed using MitoTracker Green. Unpaired Mann-Whitney test. Graphs show mean ± SD. * = p < 0.05, ** = p < 0.01, **** = p < 0.0001 (n = 50–108 fields of view from 3 biological replicates, 3 technical replicates each). (For interpretation of the references to color in this figure legend, the reader is referred to the Web version of this article.)

To corroborate our transcriptional findings and the pathways that we have identified by GSEA, we performed Western blot analysis of LC3B (and its processed forms LC3B-I and LC3B-II) and p62 as markers of autophagy and mTOR-related function, as well as of the transcription factor HIF1α, to study its stabilization, thus transcription factor activity. Metformin-treated BMDMs showed a prominent reduction of LC3B-II expression together with increased p62 levels, accountable for mTOR signalling pathway activation and autophagy inhibition [22], confirming the negative regulation of autophagy, identified at the transcriptional level (Fig. 4A). Moreover, metformin treatment resulted in a significant 1.5-fold increased expression levels of HIF1α (Fig. 4B).

Next, since HIF1α is a known inducer of the Warburg effect [23], where increased glucose uptake fuels its conversion to lactate even in the presence of oxygen, we evaluated this metabolic rewiring by quantifying metabolites in the supernatant of metformin-treated and control macrophages. Metformin-treated BMDMs showed a significant decrease of hexose and pyruvic acid, together with an increased level of L-lactic acid into the culture supernatant (Fig. 4C–Table S4). Moreover, we detected decreased levels of aspartic acid in the supernatant of metformin-treated BMDMs, which might indicate that aspartic acid is used as a source of nicotinamide adenine dinucleotide (NAD^+^) to sustain glycolysis via the malate-aspartate shuttle (Fig. 4D). Altogether, these results confirm the HIF1α-related induction of the immunologic Warburg effect [23].

In order to evaluate if metformin-associated HIF1α/mTOR interplay affected mitochondrial dynamics [24], we assessed the mitochondrial membrane potential measuring the TMRE tracer intensity and the mitochondrial morphology by live imaging. Compared with untreated cells, metformin-treated BMDMs exhibited a higher mitochondrial membrane potential as evidenced by increased TMRE staining. Additionally, they were characterized by a significantly greater number and mass of mitochondria, along with an elongated mitochondrial structure (Fig. 4E).

Collectively, these results suggest that metformin induces mTOR activation and HIF1α stabilization, thus inhibiting macrophage autophagy and inducing metabolic rewiring towards increased glycolysis and lactate production.

### 2.5 Metformin-induced HIF1α-mediated polarization occurs shortly after macrophage treatment

**Fig. 5:**
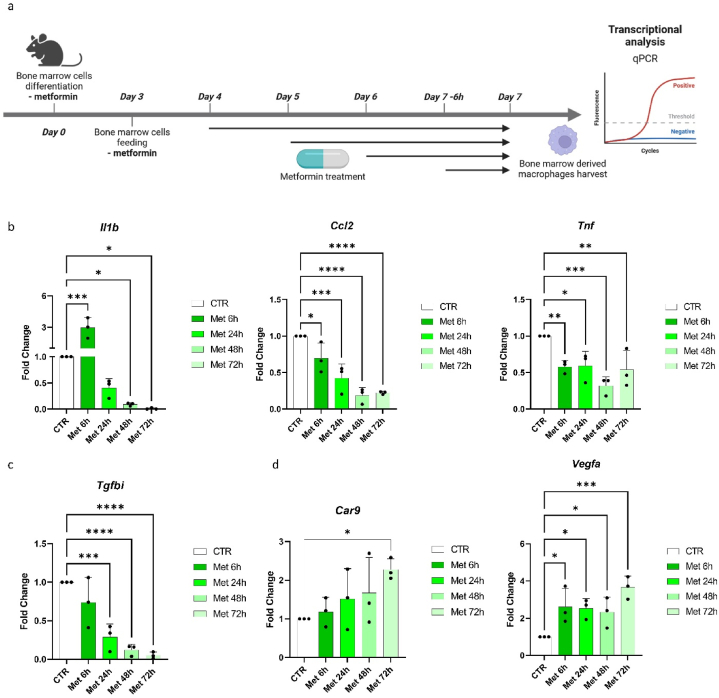
Metformin rapidly induces HIF1α activation in bone marrow cells differentiating into macrophages. **(A)** Schematic of the treatment of bone marrow myeloid cells at different time points during their differentiation towards macrophages. Bone marrow cells have been collected and differentiated with M-CSF (20 ng/mL) without metformin. Metformin (2 mM) was added either at day 4 (time point 72h), day 5 (time point 48h), day 6 (time point 24h) or day 7 for 6 h (time point 6h). Fully differentiated bone marrow-derived macrophages were collected for RNA extraction and transcriptional analysis by qPCR. **(B)** Relative quantification of mRNA expression of pro-inflammatory cytokines *Il1b*, *Ccl2* and *Tnf*. One-way ANOVA. Graphs show fold change compared to untreated BMDMs ± SD. Ns = p > 0.05, * = p < 0.05, ** = p < 0.01, *** = p < 0.001, **** = p < 0.0005 (n = 3 biological replicates). **(C)** Relative quantification of mRNA expression of *Tgfbi*. One-way ANOVA. Graphs show mean relative expression ± SD. *** = p < 0.001, **** = p < 0.0005 (n = 3 biological replicates). **(D)** Relative quantification of mRNA expression of HIF1α target genes *Car9* and *Vegfa*. One-way ANOVA. Graphs show fold change compared to untreated BMDMs ± SD. Ns = p > 0.05, * = p < 0.05, *** = p < 0.001 (n = 3 biological replicates).

Lastly, to investigate if the induction of HIF1α and the associated transcriptional and metabolic reprogramming were dependent on nutrient overtime deprivation [25], we treated bone marrow cells for 6, 24, 48 or 72 h with metformin (2 mM) during their differentiation into macrophages and analysed inflammatory and HIF1α target gene markers by qPCR (Fig. 5A). *Il1b* was upregulated after a 6 h-treatment, while it was progressively decreased at later time points. We detected a similar decrease for other pro-inflammatory genes, such as *Ccl2* and *Tnf* (Fig. 5B), and *Tgfbi* (Fig. 5C). We next evaluated the expression levels of HIF1α downstream targets *Car9* and *Vegfa*. Although the expression levels of *Car9* were significantly upregulated only after a 72-h treatment, *Vegfa* expression was already increased at early time points, suggesting a fast activation of HIF1α in BMDMs during their differentiation (Fig. 5D).

Taken together, these results provide evidence that the detected phenotype is a result of a rapid induction of HIF1α signalling as proved by the up-regulation of the expression levels of *Vegfa*, the prototypical effector of this pathway, already after a 6 h-treatment.

## 3 Discussion

Metformin, a drug commonly prescribed to diabetic patients, was previously shown to elicit protection against the development of several cancer types, such as glioma, bladder, colorectal and prostate cancer [2]. However, there remain several unanswered queries regarding the precise mechanism underlying the anticancer effects of metformin and its efficacy against different cancer types, both in diabetic and non-diabetic individuals. Considering that macrophages represent well-established mediators that precede the onset of cancer and shape its evolution [15], understanding the reaction of macrophages towards metformin exposure may explain its protective role against cancer onset and progression. In this work, we elucidated the effect of metformin on mouse bone marrow cells during their differentiation towards macrophages by characterizing their phenotypical and functional adaptation. First, we identified a change of morphology from a prototypical round shape to a more spindle-shaped form. A previously published proteomic analysis of human monocyte-derived spindle-shaped macrophages *in vitro* showed increased expression levels of endocytosis-related and heat-shock proteins [26]. Moreover, elongated macrophages have been detected in psoriasis lesions, wherein they display enhanced T cell activating functions, both antigen presentation-dependent and independent, and increased phagocytic capacity [27,28], features that we also observed in our metformin-treated macrophages. Interestingly, increase of efferocytosis has been shown to be mediated by AMPK activation upon 2-h treatment with metformin or an AMPK activator [29]. However, considering the different pathways involved in efferocytosis and phagocytosis, it is not possible to conclude that long-term metformin treatment increases the phagocytic capacity of macrophages via AMPK activation [30]. Phagocytosis has been associated with both pro-tumoural and immune-suppressive action of macrophages, but also with T cell activating and tumour-suppressive one as macrophages can activate CD8^+^ T cells and control tumour growth via cross-presentation of exogenous engulfed antigens on MHC class I molecules [31,32]. However, highly phagocytic macrophages, which also show an increase of oxidative phosphorylation, are mostly related to immune suppression and reduced overall survival in lung adenocarcinoma patients [33]. Due to this dicothomic role of phagocytosis, we studied the effect on tumour cell survival and the expression of T cell-activating molecules on macrophages. We found that metformin-treated macrophages reduced the viability of GL261 glioma cells, thus suggesting that the highly phagocytic phenotype of metformin-treated macrophages correlates with improved anti-tumoral activities. Moreover, they showed induction of CD80 expression on the cell surface unrelated to CD86 upregulation. The expression of these costimulatory molecules is related to T cell activation, proliferation and IL2 production [[34], [35], [36]]. CD80 and CD86 are both ligands of CD28, whose binding induces activation of T cells, and CTLA-4, which, on the contrary, induces suppression of T cell function. In this context, CD86 induces augmented recycling of CTLA-4, leading to prolonged activity of this immuno-regulator, while CD80, due to its higher affinity to CTLA-4, induces its ubiquitination preventing its recycling and function on the T cell membrane [37]. The preferential induction of expression of CD80 may then lead to stronger dampening of the immuno-regulatory function of CTLA-4, then potentiating anti-tumour immune responses.

It is known that CD80 expression in macrophages is related to their classical pro-inflammatory activation [38], but previous studies showed that short-term metformin treatment of differentiated macrophages modulates their pro-inflammatory features and skew them towards the M2 state via AMPK pathway activation, both *in vitro* and *in vivo* [39,40]. However, our findings contradict previous knowledge, which may be the result of the long-term exposure of bone marrow cells to the drug, which can activate alternative pathways along differentiation. It is important to mention that metformin is not metabolized in the organism, as it is eliminated in the urine as such [41]. Thus, diabetic patients under metformin treatment are continuously exposed to the drug, which may reflect the long-term exposure in our *in vitro* model.

Additionally, we found that metformin-treated BMDMs display a reduced sensitivity towards polarization stimuli, such as LPS and TGFβ. It has been previously shown that the concomitant treatment with metformin and LPS affects the classical M1-like macrophage polarization [16]. Here, we show that metformin treatment induces long-term resistance of macrophages to pro-inflammatory cytokines at the transcriptional level. Additionally, metformin-treated macrophages showed decreased expression of anti-inflammatory markers, such as *Tgfb1*, while they displayed almost complete abrogation of *Tgfbi* and *Il10* expression both at baseline and after LPS-stimulation. Similarly, TGFβ-stimulated metformin-treated macrophages displayed lack of anti-inflammatory polarization, exemplified by the absence of induction of the prototypical TGFβ target gene *Tgfbi*. Taken together, these results demonstrate that metformin-treated macrophages exhibit low polarization capacity towards pro- or anti-inflammatory states. It is interesting to note that the inhibition of the pro-inflammatory cytokine release after LPS stimulation may result *in vivo* in reduced chronic inflammatory activity of macrophages, which can then lead to reduced tumour onset [42]. For this reason, the long-term effect of metformin on macrophages in individuals affected by diabetes or obesity [43], conditions characterized by chronic inflammation [44], should be further investigated. In fact, it has been shown that monocytes collected from obese or diabetic patients are characterized by enhanced pro-inflammatory features combined with expression of M2-like macrophage markers, whereas metformin-treated diabetic patients show a monocytic phenotype comparable to healthy individuals [45]. These results suggest that metformin counteracts polarizing stimuli already at the monocytic stage of differentiation towards macrophages. Together with the previous observations, our findings suggest a long-lasting effect of metformin over the differentiation stages, thus resulting in reduced macrophage polarization plasticity.

At the transcriptional level, metformin-treated macrophages showed an enrichment of M1-signature genes concomitantly with downregulation of M2-related genes. Interestingly, it has been shown that metformin induces similar transcriptional modifications also in induced pluripotent stem cell-derived cardiac myocytes, exemplified by the induction of *HMOX1* and *MTHFD2* coupled with downregulation of *TGFBI* [46]. One of the main features of M1 macrophages is the secretion of pro-inflammatory cytokines, such as IL1β, TNF and IL12 [47]. Even though metformin-treated macrophages displayed an M1 signature enhancement, we detected a reduction of *Il1β* expression. It has been demonstrated that IL1β, despite its pro-inflammatory activity, is also involved in tumour progression and immune escape mechanisms. Specifically, IL1β activates NF-κB in tumour cells, inducing the expression of programmed death-ligand 1 (PD-L1), which then induces T cell exhaustion [48,49]. Furthermore, glioblastoma cells release CCL13 in response to IL1β secretion by tumour-associated macrophages (TAMs), leading to monocyte recruitment and their differentiation into TAMs [50].

By conducting gene set enrichment analysis, we demonstrate that metformin-treated BMDMs are characterized by the induction of negative regulation of autophagy at the gene expression level. Additionally, we show that metformin treatment induces the activation of the mTOR pathway, exemplified by the reduction of LC3B-II and increase of p62 at the protein level. Besides its role in autophagy regulation, mTOR is involved in macrophage polarization. Direct inhibition of mTOR via rapamycin administration enhances the expression of the pro-inflammatory markers CD86, CCR7 and IL6 in M1-like human monocyte-derived macrophages [51]. On the other hand, the deletion of the negative regulator of mTOR tuberous sclerosis complex1 (TSC1) is sufficient to induce a M1-like polarization of macrophages and resistance to M2 response via MAPK/ERK and mTOR related pathway, respectively [52]. Additionally, impairment of autophagy in macrophages leads to pro-inflammatory phenotypic acquisition, characterized by increased production of IL6 and TNF together with angiogenic features [53] and stabilization of MHC class I [54]. Autophagy activation induces the expression of MHC class II molecules, which can explain the reduction of MHC class II ^+^ macrophages after metformin treatment in our *in vitro* model [55]. However, it is commonly accepted that metformin directly inhibits mTOR via phosphorylation of TSC2 and RAPTOR [56,57]. Recently, it has been shown that the effect of metformin on Akt/mTOR pathway is dose-dependent. A 48-h treatment *in vivo* at the anti-diabetic dosage (0.03 mM and 0.3 mM) activates Akt/mTOR pathway, while it induces AMPK phosphorylation at higher dosage (3 mM) [58]. Consistently, Wang and colleagues found prominent differences based on metformin concentrations also at the phenotypical level, with low-dosage metformin-treated human macrophages showing M2-like features, whereas high-dosages inducing a pro-inflammatory phenotype [59]. In this context, the prolonged exposure of differentiating bone marrow cells to metformin may induce a reduction of the sensitivity to the drug, which can recapitulate the results obtained at lower dosages *in vivo*.

Metformin-treated macrophages showed also the activation of HIF1α and its downstream effectors. It has been previously showed that AMPK regulates HIF1α stabilization and transcriptional activity via its phosphorylation [60,61]. Moreover, metformin treatment of LPS-activated macrophages induces HIF1α degradation, thus impacting their pro-inflammatory activity [62]. However, HIF1α involvement in macrophage functions has still to be clarified. Additionally, HIF1α-mediated autophagy can induce the M2 signature after mitochondrial stress in macrophages, mainly demonstrated by the increased expression of Arg*1* and *Chil3* [63]. Conversely, HIF1α can activate NF-κB pathway directly and indirectly, inducing the expression of p65 or alarmin receptors, respectively [64,65], resulting in pro-inflammatory cytokine expression [[66], [67], [68]]. Moreover, HIF1α-mediated induction of glycolysis is also known to be a feature of pro-inflammatory macrophages [[68], [69]]. Here, we demonstrate that metformin-treated bone marrow cells display a metabolic switch towards glycolysis, which has been demonstrated to be correlated with a shift from M2-like towards M1-like macrophages *in vivo* as a result of metformin treatment [5]. We detected an increase of glucose consumption, high levels of lactate secreted in the medium together with a reduction of aspartate in the medium, suggesting the induction of the malate-aspartate shuttle to produce NAD^+^, which is necessary for ATP production during glycolysis [70]. Altogether, our findings confirm the induction of the immunological Warburg effect stimulated by metformin and its correlation with HIF1α transcriptional programs.

In metformin-treated BMDMs, we observed a higher mitochondrial membrane potential as well as a significantly greater number and area of mitochondria. Additionally, mitochondria were more elongated than in untreated BMDMs. Previous studies using the murine macrophage cell line RAW264.7 treated with metformin for 24 h, showed an overall improved mitochondrial respiratory efficiency and strong induction of mitofusin 2 (MFN2) protein, which is involved in mitochondrial fusion and tethering of mitochondria to the endoplasmic reticulum [71]. Accordingly, Chiche and colleagues found that long-term activation of HIF1α leads to mitochondrial fusion via MFN1 induction [72], thus suggesting that metformin-mediated mitochondrial adaptation can be mediated by HIF1α. Other studies showed that metformin protects the functional and structural integrity of mitochondria by activating AMPK, reducing dynamin related protein 1 (DRP1) expression and inhibiting DRP1-mediated mitochondrial division in diabetic patients [73]. This could explain the higher mitochondrial membrane potential as well as the larger mitochondrial mass and area that we detected in metformin-treated BMDMs. Furthermore, the mitochondrial changes observed in metformin-treated BMDMs could be a long-term adaptation to low ATP conditions induced by metformin, resulting in biogenesis of mitochondria, for example via mTOR induction, which is known to play a pivotal role in mitochondrial biogenesis and functions through the induction of the transcription factors TFAM, YY1 and PGC-1α [74].

Previous studies showed that monocytes treated with β-glucan increase their glycolytic metabolism through mTOR-HIF1α pathways. After second stimulation with β-glucan, “trained” monocytes showed stronger expression of pro-inflammatory cytokines compared to “not-trained” monocytes. This phenomenon was abrogated via inhibition of mTOR or HIF1α, as well as via induction of AMPK with 3–30 mM metformin treatment for 24 h [75]. In our study, we showed that the effect of metformin-treatment on macrophages is strongly time-dependent. In fact, short-time metformin treatment (6 h) induces an inflammatory response characterized by IL1β production and an initial induction of the HIF1α target genes *Car9* and *Vegfa*, which increases over time and is followed by a prominent reduction of the classical M1 and alternative M2 signature genes. Nevertheless, HIF1α was also shown to participate in LPS-induced immunological tolerance, characterized by the dampening of the M1 response and the enhancement of the anti-inflammatory reaction of macrophages after subsequent stimulations with LPS [76]. We show that metformin-treated BMDMs do not react by producing pro-inflammatory cytokines after LPS treatment, which would resemble the tolerant phenotype. However, they concomitantly show downregulation of anti-inflammatory cytokine gene expression, thus confuting the tolerance hypothesis. Taken together, these findings demonstrate the complex and dicothomic mechanisms of HIF1α in macrophages, leading to the possibility that its function is modulated by other co-factors.

We found that metformin-instructed bone marrow-derived macrophages displayed cytotoxic activity against glioma cells. Our observations, which shed light on the impact of metformin on crucial components of the tumour microenvironment, add weight to a number of studies supporting the use of metformin as an effective drug against gliomas, as well as its testing in clinical trials [77,78].

In summary, our study describes the impact of metformin on bone marrow-derived macrophages when treated during their differentiation from bone marrow cells at the morphological, functional and transcriptional levels. Our findings suggest that long-term treatment with metformin increases immunological activities of macrophages such as phagocytosis, T cell activation via co-stimulatory molecule expression and anti-tumour profiles. At the same time, metformin reduces both pro- and anti-inflammatory cytokine production, also after polarizing stimuli. Mechanistically, long-term exposure to metformin induces transcriptional programs related to HIF1α stabilization and mTOR activation, in contrast with the prototypical effect of metformin on AMPK activation. Taken together, our results suggest that the effect of metformin on tumour onset and progression may be related to its activity on macrophages, reducing their pro-inflammatory features and inducing an anti-tumour phenotype.

### 3.1 Limitations of the study

This is a descriptive and mechanistic study elucidating the consequences of metformin treatment in mouse bone marrow-derived macrophage cultures *in vitro*. However, further studies would need to explore if the impact of metformin on macrophages using *in vivo* tumour models is comparable to our findings *in vitro*, thus explaining the tumour growth-controlling activity of metformin.

## 4 Materials and methods

### 4.1 Mouse bone marrow-derived macrophage differentiation and treatments

C57BL/6NCrl mice were bred and maintained at the Luxembourg Centre for Systems Biomedicine (LCSB) Rodent Facility (RRID:SCR_025267) at the University of Luxembourg, at a relative humidity of 40–70 %, a temperature of 22 °C and on a 12 h light/dark cycle with provided food and water *ad libitum*. All the experiments were conducted following the national guidelines of the animal welfare law in Luxembourg (Règlement grand-ducal adopted on January 11th, 2013). The used protocol was not subjected to a project authorization by the Luxembourg national authority. However, the procedures, i.e. organ collection after cervical dislocation, fulfilled the rules and guidelines defined by the Animal Experimentation Ethics Committee of the University of Luxembourg. Bone marrow-derived macrophages (BMDMs) were differentiated as previously described [79]. Briefly, female mice between two and six months of age were euthanized via cervical dislocation and legs dissected out. Bone marrows from tibias and femurs were collected through flushing. Red blood cells (RBCs) were lysed and bone marrow cells were cultured in low-attachment conditions for 7 days in DMEM GlutaMax (Gibco/Life Technologies) supplemented with 10 % fetal bovine serum (FBS) (Gibco/Life Technologies), 1 % HEPES (Life Technologies), 100 U/mL pen-strep (Lonza), and 20 ng/mL of m-CSF (R&D Systems), with or without metformin hydrochloride (Merck). Additional complete medium was added on day 4. To evaluate the early effects of metformin, bone marrow cells were treated with or without metformin 72, 48, 24, and 6 h prior the end of the differentiation. Fully differentiated BMDMs were treated with 10 ng/mL lipopolysaccharide (LPS; Sigma-Aldrich) or 20 ng/mL murine transforming growth factor beta (TGFβ; R&D Systems).

### 4.2 Morphological analyses

Untreated or metformin-treated fully differentiated BMDMs were stained on coverslips in low-attachment conditions. Cells were fixed in 4 % PFA (Lonza) for 10 min and washed with PBS without calcium-magnesium. Cells were permeabilised and blocked, followed by a 90 min incubation with phalloidin (Cytoskeleton Inc.) at room temperature shielded from light. Coverslips were mounted on glass slides in DAPI-containing mounting medium (Invitrogen). Coverslips were imaged on a Nikon Eclipse microscope at 10× magnification for each condition in three biological replicates and analysed using CellProfiler™. Representative pictures were collected using LSM880 confocal microscope at 63× magnification (Carl Zeiss).

### 4.3 Phagocytosis assay

Fully differentiated BMDMs with and without prior metformin treatment were cultured in DMEM with 0.5 μg/mL pHrodo™ Red Phagocytosis Particles (Invitrogen) and incubated in Incucyte S3 (Sartorious). An aggregate of 36 pictures per well were acquired every hour for a duration of 25 h at 20× magnification. Phagocytosis was quantified using Incucyte 2022A (Sartorius). Red fluorescence signal was quantified applying a mask and the parameters “red object area” and “cell surface area” were extracted. Data were normalised on the phagocytic index at first time point.

### 4.4 Cytotoxicity assay

Fully differentiated BMDMs with and without prior metformin treatment were detached by scraping and replated into a 96-well plate and allowed to recover overnight. GL261 murine glioma cells were stained with CellTrace™ CFSE (Invitrogen) following the manufacturer's instructions as previously published [80]. Briefly, CFSE stock was dissolved in DMSO to obtain a solution 5 mM. Then, 1 × 10^6^ GL^26^1 cells were incubated 20 min at 37 °C with 1 mL of CFSE working solution (5uM in PBS) followed by dye quenching with DMEM + 10 % FBS + 1 % P/S. Next, CFSE-stained GL261 cells were seeded on top of BMDMs in an effector: target (BMDMs: GL261 cells) ratio of 1:1 or 2:1. After 24 h of co-culture, the supernatant and the cells have been collected via scraping. After washes, the cell mix was stained with TO-PRO-3 Iodide for dead cell identification following the manufacturer's instructions. TO-PRO-3 stock was dissolved in 1 mL of DMSO to obtain a 1 mM solution. Next, 10uM of TO-PRO-3 solution was used to stain the cells (1uL of TO-PRO-3 solution in 100uL of PBS + 0.2 % BSA). After 30 s of incubation, cells were recorded using NovoCyte Quanteon (Agilent). We analysed FCS files with FlowJo software (10.8.1) (BD).

### 4.5 Flow cytometry analyses

Fully differentiated BMDMs were scraped from the support and resuspended in PBS complemented with 0.02 % bovine serum albumin (BSA) (10.13039/100004334Merck) and then incubated with TruStain fcX (anti-mouse CD16/32) (Biolegend) to avoid non-specific binding. Then, surface marker staining was performed using Brilliant Violet 510™ anti-mouse CD80, Brilliant Violet 605™ anti-mouse CD86, PerCp/Cy5.5 anti-mouse CD11b, PE/Cyanine7 anti-mouse H-2Kb/H-2Db, APC anti-mouse I-Ab, PE anti-mouse F4/80 (Biolegend) and LIVE/DEAD™ Fixable Near-IR Dead Cell Stain Kit (Invitrogen). More information about all the antibodies used in Table S5. Cells were recorded using NovoCyte Quanteon (Agilent). We analysed FCS files with FlowJo software (10.8.1) (BD).

### 4.6 Microarray analyses (Clariom™ S)

Fully differentiated BMDMs were harvested and RNA was extracted using the RNeasy kit (Qiagen) according to the manufacturer's instructions. Total RNA quantification was performed using the Qubit Fluorometer (Thermo Scientific) and its integrity was analysed using a Fragment Analyzer system (Agilent). All RQN were above 8.5. Gene-level expression microarray-based analysis has been performed throughout mouse Clariom™ S Pico assay (ThermoFisher Scientific) according to the manufacturer's instructions.

### 4.7 Gliovis analysis

Differentially expressed genes were submitted to Gliovis online portal to evaluate the impact of specific genes on glioma patient overall survival. We took advantage of TCGA_GBMLGG dataset and optimal cut-off was determined using maximally selected rank statistic according to the application developer.

### 4.8 Gene ontology, gene set enrichment analysis (GSEA) and KEGG pathway analysis

Differentially expressed genes (log2FC ≥ ±0.5, p-value<0.05) have been submitted to ClusterProfiler R package [17,81] for Gene Ontology Biological Process analysis (GO_BP). Gene expression profiles of metformin-treated and untreated BMDMs have been submitted to GSEA 4.3.2 [18,19] following the developer guidelines. Briefly, gene expression matrix was uploaded and GO_BP and KEGG 2019 mouse gene sets were screened to identify which biological processes were upregulated (NES<0) or downregulated (NES>0) in metformin-treated BMDMs. Classical (M1) and alternative (M2) macrophage signatures have been extracted from Orecchioni et al. [21] (Table S6) and uploaded as gene sets and compared with metformin-treated macrophage signature.

### 4.9 Reverse transcription and qPCR

Fully differentiated BMDMs were harvested and RNA was extracted using the RNeasy kit (Qiagen) according to the manufacturer's instructions. Total RNA quantification was performed using the Nanodrop (Thermo Scientific). For cDNA synthesis, RNA was reverse-transcribed using SuperScript III reverse transcriptase (Invitrogen), 1 μL/reaction of oligo(dT)20 50 μM (Invitrogen) for the priming of the reaction, according to the manufacturer's instructions. The reaction was performed for 60 min at 50 °C. Diluted cDNA was then mixed with 10 μL of Fast SYBR Green Master Mix (Applied Biosystems) and 0.5 μL of 10 μM gene-specific forward and reverse primers for gene expression analysis. qPCRs were carried out in 384-well plates via QuantStudio 5 (Applied Biosystems) using the following program: 20 s at 95 °C, 35 cycles of PCR stage of 1 s at 95 °C and 20 s at 60 °C and a melt curve stage of 15 s at 95 °C, 1 min at 60 °C and 10 s at 95 °C. All samples were run in technical triplicates and the average threshold cycle (Ct) values were used to relatively quantify the product by the ΔΔCt method, using L27 ribosomal protein for normalization. Primers are listed in Table S7.

### 4.10 Western blot

Fully differentiated metformin-treated and untreated BMDMs were washed once with ice-cold PBS, harvested via scraping, and incubated in 1 x RIPA buffer (Millipore, 20–188) supplemented with protease inhibitor cocktail (Roche, 11697498001) and phosphatase inhibitor cocktail (Roche, 04906837001) for 30 min at 4 °C, with occasional vortexing (10 s, three times). Following centrifugation (16 000 g, 15 min) at 4 °C, the lysates were stored at −80 °C. Protein extracts were quantified using the BRADFORD-solution (Bio-RAD, 5000006) and heated for 10 min at 95 °C in LDS-sample loading buffer (Thermo Fisher Scientific, NP0008) containing 50 mM dithiothreitol (Amersham Biosciences, ref. 17-1318-02) before being subjected to SDS-PAGE gel electrophoresis (NuPage™ 4–12 % Bis–Tris Gel Invitrogen, NP0322box) and wet transfer O/N at 4 °C to a PVDF membrane (Thermo Fisher Scientific). The membrane was blocked in TBS containing 0.1 % TritonX-100 (TBST) and 5 % dry milk for 1 h at room temperature (RT) and incubated overnight at 4 °C with the appropriate primary antibody. Anti-HIF1α (Abcam), anti-p62 (Progen) and anti-LC3 (Cell signalling technologies) were used for protein detection while anti-α-actin (Merck Millipore) was used for signal normalization. Following three washes with TBST, the membrane was then incubated with the appropriate horseradish peroxidase (HRP)-conjugated secondary antibody in TBST containing 5 % dry milk for 1 h at RT. Following 3 washes with TBST, protein bands were detected with the ECL reagent (Super Signal West Femto Maximum Sensitivity, Thermo Fisher Scientific) according to the manufacturer's instructions and visualized by exposing the membrane to the ImageQuant LAS 4000 acquisition system (GE Healthcare). ImageJ software was used for Protein quantification (densitometry). Actin was used as loading control and normalizer. Information of all used antibodies in Table S5.

### 4.11 Metabolomics analysis

Bone marrow cells were differentiated into BMDMs as previously described with and without metformin. On day 7, BMDM supernatant was collected for metabolite extraction. Briefly, 5 μl BMDM supernatant were mixed with a solution of chloroform, methanol mix with internal standards and water in a ratio 1:3:3. After centrifugation, the aqueous polar phase was collected and submitted to liquid chromatography coupled with mass spectrometry (LC-MS) analysis. Metabolite analysis was performed by injecting 5 μl aqueous polar phase into a Vanquish® Flex UHPLC system (Thermo Scientific) coupled to a Q –Exactive® Plus Orbitrap® mass spectrometer (Thermo Scientific). Chromatography was carried out with a SeQuant® ZIC®-pHILIC 5 μm polymer 150 × 2.1 mm column (Merck AG.) protected by a SeQuant® ZIC®-pHILIC Guard 20 × 2.1 mm pre-column. The temperature of the column was kept at 45 °C and the flow rate was set to 0.2 ml/min. The mobile phases consisted of 20 mmol/L ammonium carbonate in water, pH 9.2 and supplemented with 5 μM of medronic acid (Eluent A) and Acetonitrile (Eluent B), loading and gradient occurs at 0,2 mL/min flow rate; loading step last 2min with 80 % B; then starts a linear gradient ranging from 80 % B to 20 % B in 15 min; at 18 min starts the regeneration step with 80 % B; from 20 min until 24,5 min column is re-equilibrated with 80 % B at flow rate of 0.4 mL/min 5 μL of sample were injected into the machine. The MS experiment was performed using electrospray ionization with polarity switching enabled. Source parameters were applied as written here: sheath gas flow rate, 30; aux gas flow rate, 10; sweep gas flow rate, 0; spray voltage, 4 kV (+)/3.5 kV (−); capillary temperature, 350 °C; S-lense RF level, 50; aux gas heater temperature, 100 °C. The Orbitrap mass analyzer was operated at a resolving power of 70 000 (at 200 *m*/*z*) in full-scan mode (scan range: *m*/*z* 75–1000; automatic gain control target set to 1^e6^ charges with a maximum injection time: 250 ms). Data from LC-MS measurements were acquired with the Thermo Xcalibur software (Version 4.3.73.11) and analysed using TraceFinder (Version 5.1). Relative quantification of the metabolites was obtained from the calculation of the area under the curve of the respective peaks normalised to the area under the curve of the appropriate internal standard (i.e Nε-Trifluoroacetyl-L-lysine for pyruvic acid and lactic acid, and ^13^C_10_,^15^N_5_-AMP for aspartic acid and hexoses).

### 4.12 Mitochondrial morphology and membrane potential analysis

Bone marrow cells were differentiated into BMDMs as previously described with and without metformin in a 96-well plate at day 0 (P96.1.5H-N, #1.5 High Performance Cover Glass (0.17 ± 0.005 mm), Cellvis) at a density of 1.5 x105 cells per well. Cells were plated to have 3 technical replicates per condition from 3 mice. The mitochondrial membrane potential was assessed at day 8, where cells were incubated with 150 μL/well of full medium with TMRE (Life Technologies, T669) at 10 nM at 37 °C for 30 min. Cells were co-stained with Hoechst 33342 (Life Technologies, 62249) at 1 μM and MitoTracker Green-FM (Life Technologies, M7514) at 100 nM to derive the mitochondrial mask. Then, the medium was removed and cells were washed twice following incubation in medium containing 10 nM of TMRE. Images were acquired using a 60× water immersion objective on the Yokogawa CellVoyager CV8000 High-Content Screening System, a spinning-disk confocal microscope. The following band pass filters were used: BP 445-45, BP 525-50 and BP 600-37. Z-stacks were acquired with a slicing interval of 0.4 μm and 13 fields (images) per well were obtained. Automated segmentation of structures (nuclei, mitochondria) and feature extraction e.g., mitochondrial morphometrics were performed using Matlab (version 2012a, Mathworks) following an adapted method described in Ref. [82]. Codes can be shared upon request.

### 4.13 Statistical analyses

Statistical comparison of morphological features was performed using Mann-Whitney test. For the evaluation of the phagocytic capacity, we performed a 2-way ANOVA. Statistical analysis of the cytotoxic assay was done using a paired *t*-test as experimental replicates conducted in different batches displayed different cell death efficacy. Flow cytometry and Western blot analyses were performed using an unpaired *t*-test. Statistical evaluation of qPCR results was conducted using one-way ANOVA and Tukey multiple comparison test. Statistical evaluation of mitochondrial features was done using Mann-Whitney test. Statistical analyses of Clariom S transcriptomics data was performed using limma package in R/Bioconductor (v.4.1.0). Gene set enrichments analysis (GSEA) was performed using log2FC scoring and gene sets with FDR <0.05 were considered statistically enriched.

## Data sharing statement

The Clariom S raw data have been deposited in the ArrayExpress repository (www.ncbi.nlm.nih.gov/geo/www.ebi.ac.uk/biostudies/arrayexpress) with the accession number E-MTAB 14199.

## CRediT authorship contribution statement

**Andrea Scafidi:** Writing – review & editing, Writing – original draft, Visualization, Methodology, Investigation, Formal analysis, Data curation, Conceptualization. **Frida Lind-Holm Mogensen:** Writing – review & editing, Methodology, Investigation, Formal analysis, Data curation. **Eleonora Campus:** Writing – review & editing, Methodology, Formal analysis. **Alexandros Pailas:** Writing – review & editing, Methodology, Investigation, Formal analysis. **Katrin Neumann:** Writing – review & editing, Methodology, Formal analysis. **Nathalie Legrave:** Writing – review & editing, Methodology, Formal analysis. **François Bernardin:** Writing – review & editing, Methodology, Formal analysis. **Sandro L. Pereira:** Writing – review & editing, Visualization, Methodology, Investigation, Formal analysis. **Paul M.A. Antony:** Writing – review & editing, Methodology, Investigation, Formal analysis. **Nathalie Nicot:** Writing – review & editing, Supervision, Methodology. **Michel Mittelbronn:** Writing – review & editing, Methodology, Formal analysis. **Anne Grünewald:** Writing – review & editing, Supervision. **Petr V. Nazarov:** Writing – review & editing, Visualization, Supervision, Methodology, Investigation, Formal analysis, Data curation. **Aurélie Poli:** Writing – review & editing, Methodology. **Eric Van Dyck:** Writing – review & editing, Supervision, Methodology. **Alessandro Michelucci:** Writing – review & editing, Writing – original draft, Visualization, Supervision, Resources, Project administration, Methodology, Investigation, Funding acquisition, Data curation, Conceptualization.

## Declaration of competing interest

The authors declare that the research was conducted in the absence of any commercial or financial relationships that could be construed as a potential conflict of interest.
