# Supplementary material for: Metformin impacts the differentiation of mouse bone marrow cells into macrophages affecting tumour immunity
Source: Heliyon. 2024 Sep 11;10(18):e37792. doi: 10.1016/j.heliyon.2024.e37792 (PMC11417223; doi:10.1016/j.heliyon.2024.e37792)
Supplement: Multimedia component 1 [file mmc1.docx]

**Table S1. List of DEGs between metformin-treated and untreated BMDMs (p value < 0.05).**

| Gene symbol | Log FC | P value |
| --- | --- | --- |
| Plac8 | -3,89 | 2,4E-07 |
| Mcpt8 | -4,35 | 7,7E-07 |
| Akr1c18 | -4,05 | 5,0E-06 |
| Dcn | -3,45 | 5,4E-06 |
| Mpo | -3,90 | 7,4E-06 |
| Egln3 | 3,02 | 8,2E-06 |
| Fabp3 | -2,70 | 8,9E-06 |
| Prg2 | -6,25 | 1,3E-05 |
| Prss34 | -3,66 | 1,3E-05 |
| Slco4a1 | -4,56 | 1,7E-05 |
| Dhcr24 | -4,00 | 2,4E-05 |
| Pmepa1 | -2,02 | 4,3E-05 |
| Fam213b | 1,78 | 5,3E-05 |
| Mrap | -1,52 | 5,8E-05 |
| Ctsg | -2,29 | 6,1E-05 |
| Timp1 | -2,07 | 7,0E-05 |
| Kit | -3,40 | 7,3E-05 |
| Kcne3 | -2,08 | 9,1E-05 |
| Aifm2 | 1,63 | 9,7E-05 |
| Exosc6; Aars | 1,57 | 1,1E-04 |
| Rbp1 | -1,58 | 1,1E-04 |
| Ero1l | 1,92 | 1,1E-04 |
| Fdps | -1,74 | 1,2E-04 |
| Hp | -1,65 | 1,3E-04 |
| AI506816 | -2,38 | 1,4E-04 |
| Acyp2 | -1,55 | 1,7E-04 |
| Tpbg | -1,97 | 1,7E-04 |
| Mthfd2 | 1,75 | 1,8E-04 |
| Aqp11 | 1,75 | 2,0E-04 |
| Hpgd | -3,97 | 2,3E-04 |
| Serpinb2 | -3,43 | 2,3E-04 |
| Elane | -2,53 | 2,4E-04 |
| Mmp13 | 1,38 | 2,5E-04 |
| Antxr1 | -1,85 | 2,5E-04 |
| Clybl | 1,18 | 2,9E-04 |
| Pitpnc1 | 1,19 | 2,9E-04 |
| Ak4 | 1,66 | 3,0E-04 |
| Gid4 | 1,39 | 3,1E-04 |
| Cd5l | 1,96 | 3,3E-04 |
| Cdk15 | 1,63 | 3,3E-04 |
| Enpp2 | -1,54 | 3,4E-04 |
| Spg20 | 1,70 | 3,4E-04 |
| Olfr110 | -1,68 | 3,6E-04 |
| Slc16a6 | 1,14 | 3,6E-04 |
| Phyhd1 | 2,81 | 3,7E-04 |
| Abcb1b | -1,52 | 3,8E-04 |
| Stard6 | 1,81 | 4,0E-04 |
| Spint1 | -1,62 | 4,0E-04 |
| Antxr2 | 1,56 | 4,1E-04 |
| 2510009E07Rik | -1,51 | 4,1E-04 |
| H2-Ab1 | -4,42 | 4,3E-04 |
| Penk | -1,49 | 4,3E-04 |
| Sars | 1,44 | 4,4E-04 |
| Pde7b | 1,27 | 4,4E-04 |
| Luc7l2 | 1,38 | 4,6E-04 |
| Cdkn1a | 1,52 | 4,9E-04 |
| Mtmr10 | 1,48 | 5,4E-04 |
| Tle1 | -1,47 | 5,6E-04 |
| Soat2 | 1,11 | 5,8E-04 |
| Fam118a | 1,12 | 6,1E-04 |
| Akr1c13 | 1,70 | 6,1E-04 |
| Pdp1 | 1,00 | 6,6E-04 |
| Serpinb6b | 1,24 | 7,0E-04 |
| Slc6a12 | -2,94 | 7,2E-04 |
| Vegfa | 1,15 | 7,3E-04 |
| Arrdc3 | 1,84 | 7,4E-04 |
| Zfp292 | 1,11 | 7,7E-04 |
| Fggy | 1,15 | 7,7E-04 |
| Pgk1 | 1,70 | 7,8E-04 |
| Sh2d1b1 | -2,47 | 7,9E-04 |
| Rbpj | 1,06 | 8,1E-04 |
| Htatip2 | 1,37 | 8,7E-04 |
| Sipa1l1 | 1,40 | 8,8E-04 |
| Scrn3 | 1,56 | 8,9E-04 |
| Cd74; Mir5107 | -4,10 | 8,9E-04 |
| Ypel2 | 1,33 | 8,9E-04 |
| Cdh2 | -1,37 | 9,0E-04 |
| Fads2 | -2,28 | 9,3E-04 |
| Gm11110 | 1,12 | 9,3E-04 |
| Gm10197 | 1,07 | 9,5E-04 |
| Slfn3 | 1,10 | 9,8E-04 |
| Fxyd2 | -3,54 | 9,9E-04 |
| Card11 | -1,79 | 1,0E-03 |
| Emc9 | 1,77 | 1,0E-03 |
| Rgl1 | 2,06 | 1,1E-03 |
| Ctsl | 1,31 | 1,1E-03 |
| Nfib | -1,75 | 1,1E-03 |
| Marveld1 | -1,07 | 1,1E-03 |
| Eng | 1,78 | 1,1E-03 |
| Ogn | 1,53 | 1,1E-03 |
| Adssl1 | 1,33 | 1,1E-03 |
| Gpm6b | -1,15 | 1,1E-03 |
| Sh2d1b2 | -0,95 | 1,2E-03 |
| Fasn | -2,07 | 1,2E-03 |
| Tmem176a | -2,53 | 1,2E-03 |
| D17Wsu92e | 1,02 | 1,2E-03 |
| Rasgrp4 | -1,52 | 1,2E-03 |
| Aoah | 1,38 | 1,2E-03 |
| H2-Aa | -3,08 | 1,2E-03 |
| Slc5a3; Mrps6 | -1,80 | 1,2E-03 |
| Ndrg2 | -1,31 | 1,2E-03 |
| Ell | 1,36 | 1,3E-03 |
| Pde1b | 1,49 | 1,3E-03 |
| Pcolce | -1,67 | 1,3E-03 |
| Slc25a51 | 0,90 | 1,3E-03 |
| Ly6c1 | -3,84 | 1,4E-03 |
| Postn | -1,77 | 1,4E-03 |
| Ube2h | 1,29 | 1,4E-03 |
| Pbx2 | 1,06 | 1,4E-03 |
| Zfp385a | -1,11 | 1,4E-03 |
| Rab44 | -1,07 | 1,4E-03 |
| Spata13 | -1,00 | 1,4E-03 |
| Hilpda | 1,50 | 1,4E-03 |
| Cpa3 | -2,39 | 1,5E-03 |
| Sfrp4 | -1,53 | 1,5E-03 |
| Fcrl1 | -1,92 | 1,6E-03 |
| Prelid2 | 1,84 | 1,6E-03 |
| Scd2; Mir5114 | -2,45 | 1,6E-03 |
| Cacna1b | -1,03 | 1,6E-03 |
| Bcat1 | -1,22 | 1,7E-03 |
| Napepld | 1,36 | 1,7E-03 |
| Fam92a | 1,60 | 1,7E-03 |
| Egr1 | 1,25 | 1,7E-03 |
| Slc25a37 | 1,21 | 1,7E-03 |
| Dip2c | 1,43 | 1,7E-03 |
| Muc13 | -1,09 | 1,8E-03 |
| Rin3 | -1,31 | 1,8E-03 |
| Tmem50b | -1,26 | 1,8E-03 |
| Ramp1 | -1,51 | 1,8E-03 |
| Tgfbi | -3,25 | 1,8E-03 |
| Psme4 | 1,06 | 1,8E-03 |
| Nme4 | 1,81 | 1,8E-03 |
| Hmox1 | 1,81 | 1,9E-03 |
| Slc19a2 | 1,42 | 1,9E-03 |
| Klhl42 | 0,95 | 1,9E-03 |
| Gpi1 | 1,11 | 1,9E-03 |
| Tmem68 | 1,59 | 2,0E-03 |
| Slc22a4 | 1,57 | 2,0E-03 |
| Anapc16 | -1,05 | 2,0E-03 |
| Fmnl1 | -1,41 | 2,1E-03 |
| Klrb1a | -1,68 | 2,1E-03 |
| Tmppe; Glb1 | 1,30 | 2,1E-03 |
| Cd7 | -1,50 | 2,1E-03 |
| Txndc16 | 1,14 | 2,1E-03 |
| H2-DMa | -1,42 | 2,2E-03 |
| Awat1 | -2,00 | 2,2E-03 |
| Akr1c12 | 1,24 | 2,2E-03 |
| Ly6c2 | -3,80 | 2,2E-03 |
| Usp2 | -1,33 | 2,2E-03 |
| Asns | 1,44 | 2,3E-03 |
| Mcoln3 | -2,54 | 2,3E-03 |
| Lrrc51 | 0,92 | 2,3E-03 |
| Lbr | -1,09 | 2,3E-03 |
| Gsta3 | 2,91 | 2,3E-03 |
| Nrp1; Mir1903 | 1,52 | 2,3E-03 |
| Pdk1 | 1,53 | 2,4E-03 |
| Fcer1a | -1,02 | 2,4E-03 |
| Atf5 | 0,91 | 2,4E-03 |
| Hesx1 | 1,27 | 2,4E-03 |
| Adm | 2,06 | 2,5E-03 |
| Rhoj | -1,78 | 2,5E-03 |
| Gdi1 | 0,96 | 2,5E-03 |
| Vcan | -0,89 | 2,5E-03 |
| Fem1c | 0,82 | 2,5E-03 |
| Tuba4a | -1,25 | 2,5E-03 |
| Fbxl5 | 1,11 | 2,6E-03 |
| Sept8 | 1,28 | 2,6E-03 |
| Sptbn1 | 1,33 | 2,6E-03 |
| Pgm1 | 0,97 | 2,6E-03 |
| Kif23 | -1,41 | 2,6E-03 |
| Hnrnph1 | -1,06 | 2,7E-03 |
| Dcun1d3 | 1,30 | 2,7E-03 |
| Ear1 | -1,63 | 2,7E-03 |
| D17H6S56E-5 | -2,41 | 2,7E-03 |
| Bex6 | -1,20 | 2,7E-03 |
| Sympk | 0,92 | 2,7E-03 |
| Tmsb15b2; Tmsb15b1; Tmsb15l | -1,41 | 2,7E-03 |
| Sumo3 | -0,89 | 2,7E-03 |
| Ccdc120 | -0,83 | 2,8E-03 |
| Gpr162 | -1,15 | 2,8E-03 |
| Pla2g4a | -0,88 | 2,8E-03 |
| Cox15 | 0,84 | 2,8E-03 |
| Tmem9 | 0,90 | 2,8E-03 |
| Wipi1 | 0,89 | 2,8E-03 |
| Ikbkg | 1,91 | 2,8E-03 |
| Bcar3 | 1,04 | 2,9E-03 |
| Cd34 | -1,17 | 2,9E-03 |
| Cyp4f18 | -2,11 | 2,9E-03 |
| Rhbdd1 | 1,23 | 3,0E-03 |
| Cbx4 | 1,25 | 3,0E-03 |
| Olfr111 | -0,99 | 3,0E-03 |
| Asah2 | 1,00 | 3,0E-03 |
| Adgrg3 | -1,10 | 3,0E-03 |
| Adpgk | 0,90 | 3,0E-03 |
| Snx29 | 1,03 | 3,0E-03 |
| Ppil4 | 0,93 | 3,1E-03 |
| Zeb2; Mir5129 | 0,95 | 3,1E-03 |
| Rhoq | -0,86 | 3,1E-03 |
| Rnf180 | -0,84 | 3,2E-03 |
| Id3 | -2,84 | 3,2E-03 |
| Khnyn | 0,96 | 3,2E-03 |
| Gpcpd1 | 0,95 | 3,2E-03 |
| Pgrmc1 | 0,87 | 3,2E-03 |
| Lpcat1 | -0,90 | 3,3E-03 |
| Thbs1 | 1,80 | 3,3E-03 |
| Mcfd2 | 1,35 | 3,3E-03 |
| Tbc1d31 | 1,29 | 3,3E-03 |
| Gpr141 | 1,30 | 3,3E-03 |
| Rnf19a; Mir8097 | 1,02 | 3,3E-03 |
| Csde1 | 0,96 | 3,4E-03 |
| Olfm1 | -1,45 | 3,4E-03 |
| H2-Eb1 | -3,17 | 3,4E-03 |
| Ints6 | 0,82 | 3,4E-03 |
| Csnk1g1 | 1,07 | 3,5E-03 |
| Fads1 | -0,89 | 3,5E-03 |
| Tm4sf1 | -1,85 | 3,6E-03 |
| Zfp781 | 1,11 | 3,6E-03 |
| App | 0,87 | 3,7E-03 |
| Xpot | 0,77 | 3,7E-03 |
| Bbs9 | 1,03 | 3,7E-03 |
| Lyplal1 | 0,98 | 3,8E-03 |
| Hk2 | 1,74 | 3,8E-03 |
| Ccr5 | 1,18 | 3,8E-03 |
| Tmem47 | -1,44 | 3,9E-03 |
| Cul7 | 0,99 | 3,9E-03 |
| Grem1 | -2,65 | 3,9E-03 |
| Bbx | 1,07 | 3,9E-03 |
| Gpr19 | 0,90 | 3,9E-03 |
| Arg2 | -1,49 | 3,9E-03 |
| Gm8374 | -1,04 | 3,9E-03 |
| Cyp2j6 | 0,91 | 4,0E-03 |
| Cd83 | -0,84 | 4,0E-03 |
| Lonrf3 | 0,84 | 4,1E-03 |
| Scd1 | -1,66 | 4,1E-03 |
| Slc28a2 | 2,16 | 4,1E-03 |
| Layn | 1,42 | 4,1E-03 |
| Sigmar1 | -1,10 | 4,2E-03 |
| Ptger2 | 1,80 | 4,2E-03 |
| Nicn1 | 0,97 | 4,2E-03 |
| Rarres1 | -0,81 | 4,3E-03 |
| Cds1 | -1,15 | 4,3E-03 |
| Gstm2 | 2,00 | 4,3E-03 |
| Pcp4l1 | -1,12 | 4,4E-03 |
| Ugt1a2; Ugt1a6a; Ugt1a6b; Ugt1a10; Ugt1a7c; Ugt1a5; Ugt1a9; Ugt1a1 | 0,86 | 4,4E-03 |
| Lpar5 | -1,67 | 4,4E-03 |
| Mfsd6 | -1,78 | 4,4E-03 |
| Scamp1 | 1,08 | 4,5E-03 |
| Ptgs2 | -0,95 | 4,5E-03 |
| Ppid | 0,86 | 4,5E-03 |
| Sfmbt1 | 1,34 | 4,5E-03 |
| Lyrm5 | 1,36 | 4,5E-03 |
| Slc7a1 | 1,01 | 4,5E-03 |
| Sp3 | 0,86 | 4,6E-03 |
| Ckap2l | -1,28 | 4,6E-03 |
| Ccr2 | -3,11 | 4,6E-03 |
| Spats2 | 1,45 | 4,6E-03 |
| Arap3; Mir6981 | -2,04 | 4,6E-03 |
| Ube2w | 1,28 | 4,7E-03 |
| Tap1 | 0,91 | 4,7E-03 |
| 3110057O12Rik; Gm2011 | 0,99 | 4,7E-03 |
| Cks1b | -1,41 | 4,7E-03 |
| Trim16 | -0,88 | 4,7E-03 |
| Esco2 | -1,21 | 4,7E-03 |
| Gys1 | 1,51 | 4,8E-03 |
| Tmem243 | -1,16 | 4,8E-03 |
| Tsku | 1,13 | 4,9E-03 |
| Dcstamp | -2,95 | 4,9E-03 |
| Baiap2 | 1,44 | 4,9E-03 |
| Akr1b8 | 0,71 | 4,9E-03 |
| Slc17a9 | 0,76 | 4,9E-03 |
| Idh2 | 0,77 | 4,9E-03 |
| Mavs | 0,82 | 5,0E-03 |
| Gtpbp2 | 1,10 | 5,0E-03 |
| Tfb1m | 0,90 | 5,0E-03 |
| Bnip3 | 1,29 | 5,0E-03 |
| Rasgrp3 | -1,76 | 5,0E-03 |
| Suco | 1,08 | 5,0E-03 |
| Plpp1 | -1,67 | 5,0E-03 |
| Qpct | -1,33 | 5,1E-03 |
| Lrrc32 | -1,13 | 5,1E-03 |
| Myl10 | -1,58 | 5,1E-03 |
| Cald1 | -1,43 | 5,2E-03 |
| Blzf1 | 0,85 | 5,2E-03 |
| Txndc12 | 0,93 | 5,2E-03 |
| Sepn1 | 1,70 | 5,3E-03 |
| Cyp2r1 | -1,04 | 5,3E-03 |
| Gdf15 | 3,15 | 5,3E-03 |
| Dync1li2 | 1,02 | 5,3E-03 |
| Map3k2 | 1,06 | 5,3E-03 |
| Srd5a1 | -1,04 | 5,4E-03 |
| P4ha1 | 1,07 | 5,4E-03 |
| Gpx3 | -2,07 | 5,5E-03 |
| B4galt6 | 0,95 | 5,5E-03 |
| Cd244 | -0,84 | 5,5E-03 |
| Metap1 | 0,80 | 5,5E-03 |
| Jchain | -1,66 | 5,6E-03 |
| Arhgef37 | -1,20 | 5,6E-03 |
| Chd7 | -0,75 | 5,6E-03 |
| Plxdc2 | -2,35 | 5,7E-03 |
| Matk | -1,51 | 5,7E-03 |
| Stfa2 | -0,94 | 5,7E-03 |
| Utrn | 1,13 | 5,7E-03 |
| Tmem5; Gm9079 | 0,74 | 5,8E-03 |
| Cxcl16 | -0,93 | 5,8E-03 |
| Tspan3 | 0,93 | 5,8E-03 |
| Zfp445 | 1,52 | 5,8E-03 |
| Cd209a | -1,57 | 5,9E-03 |
| Actg2 | -1,40 | 5,9E-03 |
| Copz2 | 0,98 | 5,9E-03 |
| Spp1 | 1,07 | 5,9E-03 |
| Cd200r1 | 0,79 | 5,9E-03 |
| Depdc1a | -1,15 | 6,0E-03 |
| Il18 | 1,21 | 6,0E-03 |
| Sntb2 | 0,76 | 6,0E-03 |
| 9230113P08Rik | -0,93 | 6,1E-03 |
| Slc40a1 | 2,23 | 6,1E-03 |
| Nedd4 | -0,79 | 6,1E-03 |
| Clec2f | 1,29 | 6,1E-03 |
| Arl14ep | 0,87 | 6,1E-03 |
| Vmp1; Mir21a | 1,19 | 6,1E-03 |
| Afg3l2 | 1,08 | 6,2E-03 |
| Noa1 | -1,03 | 6,2E-03 |
| Mta1 | 0,68 | 6,2E-03 |
| Ankrd37 | 2,10 | 6,3E-03 |
| Coq3 | 0,71 | 6,4E-03 |
| Osbpl1a | 0,91 | 6,4E-03 |
| Tor3a | -1,07 | 6,5E-03 |
| Srpr | 0,88 | 6,6E-03 |
| Wdr91 | -0,80 | 6,6E-03 |
| Cd302 | -0,98 | 6,7E-03 |
| Ralgps2 | 1,10 | 6,7E-03 |
| Fkbp15 | 0,93 | 6,7E-03 |
| Zmynd15 | -1,28 | 6,7E-03 |
| Gtf3c3 | 0,75 | 6,8E-03 |
| Zfp729b | 0,71 | 6,8E-03 |
| Chaf1a | -1,00 | 6,9E-03 |
| Eif4a2; Snord2; Snora81 | 1,28 | 6,9E-03 |
| Ap3m1 | 0,69 | 6,9E-03 |
| Esyt2 | 0,99 | 6,9E-03 |
| B3glct | -1,03 | 6,9E-03 |
| Plk2 | 0,71 | 6,9E-03 |
| Kdelc2 | 1,05 | 7,0E-03 |
| Ppfibp2 | 0,93 | 7,0E-03 |
| Rab3il1 | -1,58 | 7,0E-03 |
| Nisch | 1,15 | 7,1E-03 |
| Cdk6 | 1,11 | 7,1E-03 |
| Mtmr3 | 0,78 | 7,1E-03 |
| Rag1; B230118H07Rik | 0,88 | 7,1E-03 |
| Aqp9 | 1,52 | 7,1E-03 |
| Tatdn3 | 0,68 | 7,2E-03 |
| Serpinb6c | 0,96 | 7,3E-03 |
| Gmpr | 1,05 | 7,3E-03 |
| Cd207 | -0,89 | 7,4E-03 |
| Plppr2 | 0,80 | 7,5E-03 |
| Nit1 | 0,93 | 7,5E-03 |
| Idi1 | -1,62 | 7,6E-03 |
| Nucb1 | 1,11 | 7,7E-03 |
| Fpr2; Fpr3 | -0,93 | 7,7E-03 |
| Cx3cr1 | -1,54 | 7,8E-03 |
| Eif2d | 0,84 | 7,8E-03 |
| Usp22 | 0,93 | 7,9E-03 |
| Gstm4 | 1,51 | 8,0E-03 |
| Sqle | -1,00 | 8,0E-03 |
| Adss | 0,80 | 8,0E-03 |
| Gpr183 | -1,15 | 8,0E-03 |
| Tnrc6a | 0,76 | 8,0E-03 |
| E2f7 | -0,73 | 8,1E-03 |
| Me1 | -1,15 | 8,2E-03 |
| Zfp72 | 1,20 | 8,2E-03 |
| Klk1b11 | -1,13 | 8,2E-03 |
| Dnaja3 | 0,66 | 8,3E-03 |
| Tyms | -0,77 | 8,4E-03 |
| Tk2 | 0,82 | 8,4E-03 |
| Cep128 | -1,03 | 8,4E-03 |
| Cd33 | -0,95 | 8,4E-03 |
| Tapt1 | 0,97 | 8,5E-03 |
| Pcnx | 1,35 | 8,5E-03 |
| Bst1 | 1,85 | 8,6E-03 |
| Sorl1 | -0,99 | 8,6E-03 |
| Cst7 | -0,85 | 8,6E-03 |
| Tmem176b | -2,95 | 8,6E-03 |
| Rabgap1l | -0,93 | 8,6E-03 |
| Htra4 | -1,21 | 8,7E-03 |
| Zfp219 | 0,74 | 8,7E-03 |
| Arl1 | 0,81 | 8,7E-03 |
| Pstpip1 | 0,77 | 8,7E-03 |
| Mgea5 | 0,73 | 8,7E-03 |
| Kbtbd11 | -0,97 | 8,7E-03 |
| Slamf8 | -1,80 | 8,8E-03 |
| Trappc6a | 1,28 | 8,8E-03 |
| Map2 | -0,65 | 8,8E-03 |
| Prtn3 | -0,81 | 8,8E-03 |
| Ndc80 | -1,22 | 8,9E-03 |
| Napa | 0,83 | 8,9E-03 |
| Cep104 | 1,00 | 8,9E-03 |
| Camkk2 | -1,11 | 8,9E-03 |
| Fndc3b | 1,11 | 9,0E-03 |
| Zfp949 | 1,04 | 9,0E-03 |
| Slc39a7 | 1,15 | 9,0E-03 |
| Usp3 | 0,88 | 9,1E-03 |
| Senp1 | -1,00 | 9,1E-03 |
| Itgb5 | -0,74 | 9,1E-03 |
| Bambi | 1,30 | 9,2E-03 |
| Ank; Mir7117 | -1,07 | 9,2E-03 |
| Glb1l | 0,76 | 9,2E-03 |
| Lman2 | 1,14 | 9,3E-03 |
| Lat | -1,14 | 9,3E-03 |
| Aldh6a1 | 0,86 | 9,4E-03 |
| Ddr2 | -0,89 | 9,4E-03 |
| Gpr35 | 1,89 | 9,4E-03 |
| Fcrls | -1,07 | 9,5E-03 |
| P4ha2 | 0,72 | 9,5E-03 |
| Mtm1 | 0,72 | 9,6E-03 |
| Apbb2 | -0,74 | 9,6E-03 |
| Rnf170 | 0,83 | 9,6E-03 |
| Sell | -2,37 | 9,6E-03 |
| Echdc1 | -0,83 | 9,6E-03 |
| Usp48 | 0,72 | 9,6E-03 |
| Siglech | -1,14 | 9,6E-03 |
| Gnpda2 | 0,68 | 9,7E-03 |
| Tas1r3 | 0,73 | 9,8E-03 |
| Lman2l | 1,05 | 9,8E-03 |
| H2afx | -0,96 | 9,8E-03 |
| Aqp1 | -0,86 | 9,8E-03 |
| Ifi27l2a | 1,38 | 9,9E-03 |
| Rassf8 | 0,91 | 9,9E-03 |
| Derl1 | 0,97 | 9,9E-03 |
| Aldh1b1 | 0,71 | 9,9E-03 |
| Yars | 0,63 | 1,0E-02 |
| Serpinb9 | 0,96 | 1,0E-02 |
| Hgsnat | -1,30 | 1,0E-02 |
| Jade3 | 1,18 | 1,0E-02 |
| Alkbh5 | 1,12 | 1,0E-02 |
| Utp6 | 0,69 | 1,0E-02 |
| Rnase12 | -1,21 | 1,0E-02 |
| C1galt1 | 0,77 | 1,0E-02 |
| Ccna2 | -1,42 | 1,0E-02 |
| Dock7 | 0,97 | 1,0E-02 |
| Lysmd4 | 0,68 | 1,0E-02 |
| Znhit3 | 0,66 | 1,0E-02 |
| Clec2d | 0,82 | 1,0E-02 |
| Slc25a36 | 0,92 | 1,0E-02 |
| Zc3h14 | 0,82 | 1,0E-02 |
| Pja2 | 0,93 | 1,0E-02 |
| Vars | 1,03 | 1,1E-02 |
| Hmgb2 | -0,93 | 1,1E-02 |
| Pygl | 1,13 | 1,1E-02 |
| Cln3 | -0,69 | 1,1E-02 |
| Pdcd4 | -0,92 | 1,1E-02 |
| Ifitm1 | -1,06 | 1,1E-02 |
| Kif13b | 0,78 | 1,1E-02 |
| Prkra | 1,01 | 1,1E-02 |
| Prmt2; Mir678 | 0,96 | 1,1E-02 |
| Cenpm | -0,80 | 1,1E-02 |
| Rubcn | 1,84 | 1,1E-02 |
| Tanc2 | 1,19 | 1,1E-02 |
| Tbc1d2 | 0,72 | 1,1E-02 |
| Ppp1r3d | -0,69 | 1,1E-02 |
| Pgpep1 | 0,81 | 1,1E-02 |
| Ice1 | 0,85 | 1,1E-02 |
| Ube2t | -0,80 | 1,1E-02 |
| E2f8 | -1,80 | 1,1E-02 |
| Prdx3 | 0,94 | 1,1E-02 |
| Eaf1 | 0,78 | 1,1E-02 |
| Lpl | 1,05 | 1,1E-02 |
| Ly6g | 0,82 | 1,1E-02 |
| Galns | 1,23 | 1,1E-02 |
| Zfp935 | 0,72 | 1,1E-02 |
| Dus3l | -1,04 | 1,1E-02 |
| Fam73a | -0,89 | 1,1E-02 |
| Tbc1d7 | 0,96 | 1,1E-02 |
| Stfa2l1 | -1,39 | 1,1E-02 |
| Alox5 | -0,89 | 1,1E-02 |
| Bhlhe40 | -1,06 | 1,1E-02 |
| Rnf114 | 0,63 | 1,1E-02 |
| Ly6a | -0,99 | 1,1E-02 |
| Olfr482 | -1,34 | 1,1E-02 |
| Ezh2 | -0,73 | 1,1E-02 |
| Gmip | -1,14 | 1,1E-02 |
| Ccbl2 | -0,85 | 1,1E-02 |
| Rassf4 | -0,94 | 1,1E-02 |
| Ppp1r3b | 0,71 | 1,1E-02 |
| Sfpq | -0,70 | 1,1E-02 |
| Hbb-bs; Hbb-b1 | 1,53 | 1,1E-02 |
| P2rx4 | 1,26 | 1,1E-02 |
| Ggt5 | 0,95 | 1,1E-02 |
| Ak1 | 1,27 | 1,1E-02 |
| Stxbp4 | 1,01 | 1,2E-02 |
| Tmem65 | 0,84 | 1,2E-02 |
| Ccne2 | -1,95 | 1,2E-02 |
| Ms4a4d | -0,62 | 1,2E-02 |
| Ethe1 | 0,72 | 1,2E-02 |
| Atp6v1h | 0,81 | 1,2E-02 |
| Prrx1 | -1,14 | 1,2E-02 |
| Kcmf1 | 0,73 | 1,2E-02 |
| Ulbp1 | 1,38 | 1,2E-02 |
| Stk19 | -0,86 | 1,2E-02 |
| Acadvl | 0,76 | 1,2E-02 |
| Irx3 | -0,93 | 1,2E-02 |
| Ccnb2 | -1,13 | 1,2E-02 |
| Dnajb13 | 0,77 | 1,2E-02 |
| Cdc42ep3 | -0,81 | 1,2E-02 |
| Enoph1 | -0,81 | 1,2E-02 |
| Adap2 | 0,80 | 1,2E-02 |
| Tpd52 | 0,61 | 1,2E-02 |
| Pgap2 | 0,67 | 1,2E-02 |
| Kdelr1 | 1,01 | 1,2E-02 |
| Ube2e1 | 0,75 | 1,2E-02 |
| Zfp110 | 0,71 | 1,2E-02 |
| Aes | 0,66 | 1,2E-02 |
| Slpi; Mir7678 | -1,35 | 1,2E-02 |
| Fkbpl | 0,74 | 1,2E-02 |
| Ly75 | -1,20 | 1,2E-02 |
| Phkb | 1,22 | 1,2E-02 |
| Amd2 | -0,81 | 1,2E-02 |
| E2f2 | -0,92 | 1,2E-02 |
| Coro2b | 0,95 | 1,2E-02 |
| Phlda3 | 1,84 | 1,2E-02 |
| Arl5c | -0,75 | 1,3E-02 |
| Map3k1 | 1,07 | 1,3E-02 |
| Nob1 | -0,63 | 1,3E-02 |
| Mbd3l1 | 0,66 | 1,3E-02 |
| Scgb1b20 | -0,71 | 1,3E-02 |
| Nus1 | 1,00 | 1,3E-02 |
| Zdhhc20 | 0,69 | 1,3E-02 |
| Metrnl | 0,66 | 1,3E-02 |
| Tstd2 | 0,69 | 1,3E-02 |
| Gss | 0,91 | 1,3E-02 |
| Gm2002; Il11ra2 | -0,95 | 1,3E-02 |
| Hgf | -0,85 | 1,3E-02 |
| Chmp7 | 0,96 | 1,3E-02 |
| Fbxo42 | 1,38 | 1,3E-02 |
| Lsm4 | -0,72 | 1,3E-02 |
| Adck4 | 0,70 | 1,3E-02 |
| Rabl6 | 0,68 | 1,3E-02 |
| Bphl | 0,73 | 1,3E-02 |
| Arnt | 0,91 | 1,3E-02 |
| Hist1h3i | -0,92 | 1,3E-02 |
| Catsperg1 | 0,87 | 1,3E-02 |
| Ap5m1 | 0,83 | 1,3E-02 |
| Rlf | 1,02 | 1,3E-02 |
| Gm4924 | 0,97 | 1,3E-02 |
| Rab13 | 0,63 | 1,3E-02 |
| Arntl | -0,80 | 1,3E-02 |
| 4930562F07Rik | -0,71 | 1,3E-02 |
| Mr1 | 1,26 | 1,3E-02 |
| Pacs1 | 0,70 | 1,3E-02 |
| Mn1 | -0,67 | 1,3E-02 |
| Car11 | 1,01 | 1,3E-02 |
| Nudt6 | -0,77 | 1,3E-02 |
| Pf4 | 1,58 | 1,3E-02 |
| Gnai3 | 0,71 | 1,3E-02 |
| Il11ra1 | -0,80 | 1,3E-02 |
| Tspan32 | 1,09 | 1,3E-02 |
| Scarf1 | 1,46 | 1,3E-02 |
| Ube2q2 | 0,94 | 1,3E-02 |
| Npm3 | -0,90 | 1,3E-02 |
| Tox4 | 0,66 | 1,3E-02 |
| Cep85l | 1,09 | 1,3E-02 |
| Epb41l1 | 1,34 | 1,4E-02 |
| Fam213a | -0,98 | 1,4E-02 |
| Aldh7a1 | 0,72 | 1,4E-02 |
| Ublcp1 | -0,77 | 1,4E-02 |
| Mdm2 | 0,74 | 1,4E-02 |
| Gabpb1 | 1,51 | 1,4E-02 |
| Hist1h4d | -1,11 | 1,4E-02 |
| Hspa9 | 0,87 | 1,4E-02 |
| Haus2 | 0,71 | 1,4E-02 |
| Cbwd1 | 0,80 | 1,4E-02 |
| Tmem150b | -1,34 | 1,4E-02 |
| Pvr | 0,71 | 1,4E-02 |
| Ccdc69 | -0,81 | 1,4E-02 |
| Trp53inp1 | 1,54 | 1,4E-02 |
| Med13 | 0,70 | 1,4E-02 |
| Lonp1 | 0,94 | 1,4E-02 |
| Fgr | -1,02 | 1,4E-02 |
| Sparc | -1,22 | 1,4E-02 |
| Ranbp1 | -0,98 | 1,4E-02 |
| Setx | 0,75 | 1,4E-02 |
| Mrps10 | 0,99 | 1,5E-02 |
| Plscr1 | -0,65 | 1,5E-02 |
| Inpp5f | 0,97 | 1,5E-02 |
| Nop16 | -1,05 | 1,5E-02 |
| Zfp719 | -0,99 | 1,5E-02 |
| Fam102a | -0,77 | 1,5E-02 |
| Polr2h | -1,04 | 1,5E-02 |
| Ptgr1 | 1,00 | 1,5E-02 |
| Casp3 | 1,04 | 1,5E-02 |
| Cryab | -0,86 | 1,5E-02 |
| Pfkl | 1,50 | 1,5E-02 |
| Hist1h2ak | -1,36 | 1,5E-02 |
| Nup160 | 1,04 | 1,5E-02 |
| Tspan7 | -1,69 | 1,5E-02 |
| Defb30 | -0,86 | 1,5E-02 |
| Taok1 | 0,82 | 1,5E-02 |
| Sesn2 | 1,71 | 1,5E-02 |
| Mapk6 | 0,74 | 1,5E-02 |
| Six1 | -0,89 | 1,5E-02 |
| Uap1 | 0,93 | 1,5E-02 |
| Ing2 | 0,90 | 1,5E-02 |
| Fam83d | -1,12 | 1,5E-02 |
| Gpr107 | 0,71 | 1,5E-02 |
| Vsig8 | -0,70 | 1,5E-02 |
| Rras | 0,88 | 1,5E-02 |
| Wdsub1 | -1,09 | 1,5E-02 |
| Ttc14 | 0,72 | 1,5E-02 |
| Fam160a2 | 0,59 | 1,5E-02 |
| Siglecf | -1,76 | 1,5E-02 |
| Zfp369 | 0,84 | 1,5E-02 |
| AI987944 | 0,75 | 1,5E-02 |
| Txnl4a | 0,62 | 1,5E-02 |
| Cd200r3 | -1,14 | 1,6E-02 |
| Fndc3a | 0,85 | 1,6E-02 |
| Spn | -0,77 | 1,6E-02 |
| Zcchc24 | 0,82 | 1,6E-02 |
| Plekhn1 | 1,52 | 1,6E-02 |
| Med20; Usp49 | 0,93 | 1,6E-02 |
| Sec24c | 0,89 | 1,6E-02 |
| Cenpp | -0,62 | 1,6E-02 |
| Eif2ak4 | 0,75 | 1,6E-02 |
| Ednrb | 1,47 | 1,6E-02 |
| Vmn1r221 | -0,78 | 1,6E-02 |
| Havcr2 | 1,14 | 1,6E-02 |
| Btbd1 | 0,86 | 1,6E-02 |
| Prr11 | -1,10 | 1,6E-02 |
| Mfsd8 | 0,86 | 1,6E-02 |
| Traip | -0,71 | 1,6E-02 |
| Phf11a | 0,65 | 1,6E-02 |
| Jak1 | 0,69 | 1,6E-02 |
| Mmp3 | -1,36 | 1,6E-02 |
| Cd276 | -1,24 | 1,6E-02 |
| Sv2a | -0,71 | 1,6E-02 |
| Emc1 | 1,18 | 1,6E-02 |
| Zfp958 | 1,41 | 1,6E-02 |
| Tuba1b | -0,85 | 1,6E-02 |
| Zfp770 | -0,96 | 1,6E-02 |
| Il1b | -1,38 | 1,6E-02 |
| Coprs | -0,69 | 1,6E-02 |
| Crtap | 0,71 | 1,6E-02 |
| Pkn1 | 0,58 | 1,6E-02 |
| Cdv3 | 0,61 | 1,6E-02 |
| Nif3l1 | -0,71 | 1,6E-02 |
| Arl6 | 0,89 | 1,6E-02 |
| C3 | -1,06 | 1,6E-02 |
| Foxred2 | 0,57 | 1,6E-02 |
| Epdr1 | -0,69 | 1,6E-02 |
| Ap4s1 | -0,82 | 1,6E-02 |
| Fes | -1,70 | 1,6E-02 |
| Trp53bp2 | 0,72 | 1,7E-02 |
| Dpf2 | 0,97 | 1,7E-02 |
| Sc5d | -0,84 | 1,7E-02 |
| Dhrs4 | -0,69 | 1,7E-02 |
| Npc1 | 0,93 | 1,7E-02 |
| Tmem260 | 0,75 | 1,7E-02 |
| Osbpl2 | 0,70 | 1,7E-02 |
| Mpp1 | 0,75 | 1,7E-02 |
| Dpep1 | -0,72 | 1,7E-02 |
| Nfkbia | -1,06 | 1,7E-02 |
| Abcc1 | 0,97 | 1,7E-02 |
| Bank1 | 1,18 | 1,7E-02 |
| Ebi3 | 1,08 | 1,7E-02 |
| Il1r2 | -0,98 | 1,7E-02 |
| Rab1b | -0,70 | 1,7E-02 |
| Dync2li1 | 0,78 | 1,7E-02 |
| Mtss1l | -0,85 | 1,7E-02 |
| Cd80 | 0,97 | 1,7E-02 |
| Diaph3 | -1,57 | 1,7E-02 |
| Hpse | 1,04 | 1,7E-02 |
| Fbxl20 | 1,00 | 1,7E-02 |
| Gm20831; Ssty1 | -0,61 | 1,7E-02 |
| Myo9b | 0,57 | 1,7E-02 |
| Arhgef12 | 0,88 | 1,7E-02 |
| Hn1l | -0,83 | 1,7E-02 |
| Snx6 | 0,67 | 1,7E-02 |
| Zbtb1 | -0,74 | 1,7E-02 |
| Prkab2 | 0,82 | 1,7E-02 |
| Gmpr2 | -0,76 | 1,7E-02 |
| Wwp1 | 0,95 | 1,7E-02 |
| Nup35 | -0,81 | 1,7E-02 |
| Tm9sf4 | 1,09 | 1,7E-02 |
| St18 | -1,09 | 1,7E-02 |
| Gm2518 | -0,88 | 1,7E-02 |
| Scimp | -1,29 | 1,7E-02 |
| Cxcr2 | -1,06 | 1,8E-02 |
| Cog2 | 1,10 | 1,8E-02 |
| Xdh | 0,72 | 1,8E-02 |
| Slc43a2 | 1,13 | 1,8E-02 |
| Cp | -0,78 | 1,8E-02 |
| Glce; Mir5133 | 0,70 | 1,8E-02 |
| Zfp729a | 0,79 | 1,8E-02 |
| Pnpla7 | -0,90 | 1,8E-02 |
| Btbd2 | 0,61 | 1,8E-02 |
| Cd55 | 1,23 | 1,8E-02 |
| Rdh10 | -0,75 | 1,8E-02 |
| Tfip11 | 0,74 | 1,8E-02 |
| Eprs | 0,79 | 1,8E-02 |
| P2ry14; F630111L10Rik | -1,42 | 1,8E-02 |
| Ugp2 | 0,70 | 1,8E-02 |
| Cdk5rap2 | 0,98 | 1,8E-02 |
| Cers2 | 0,70 | 1,8E-02 |
| Tlr7 | -0,73 | 1,8E-02 |
| Mdp1 | 0,62 | 1,8E-02 |
| Mmaa | 0,84 | 1,8E-02 |
| Smarca2 | -0,90 | 1,8E-02 |
| Tsga10 | 0,78 | 1,8E-02 |
| Tkfc | 0,63 | 1,8E-02 |
| Slc35f5 | 1,03 | 1,8E-02 |
| Pak1 | -1,21 | 1,8E-02 |
| Trnt1 | 0,84 | 1,8E-02 |
| Fnip2 | 1,07 | 1,9E-02 |
| Lama3 | 0,72 | 1,9E-02 |
| Adk | 0,66 | 1,9E-02 |
| Pfkm | 0,56 | 1,9E-02 |
| Pigk | 0,76 | 1,9E-02 |
| March1 | -0,81 | 1,9E-02 |
| Tlr8 | -0,72 | 1,9E-02 |
| Leng8 | 0,73 | 1,9E-02 |
| Fbxo6 | 0,77 | 1,9E-02 |
| Vprbp | 0,56 | 1,9E-02 |
| Pnpla8; Gm2027 | 0,79 | 1,9E-02 |
| Srgap2 | 0,61 | 1,9E-02 |
| Dock5 | -1,00 | 1,9E-02 |
| Cox6b2 | -1,39 | 1,9E-02 |
| Suds3 | 0,77 | 2,0E-02 |
| Rwdd2a | 0,79 | 2,0E-02 |
| Scmh1 | 0,61 | 2,0E-02 |
| Acaca | -0,84 | 2,0E-02 |
| Mfap3l | 0,80 | 2,0E-02 |
| Clec2i | 1,74 | 2,0E-02 |
| Hbb-bt; Hbb-b2 | 1,15 | 2,0E-02 |
| Xpo1 | 0,77 | 2,0E-02 |
| Cpq | 0,72 | 2,0E-02 |
| Olfml3 | -1,20 | 2,0E-02 |
| Wnt5a | -0,69 | 2,0E-02 |
| Mapkapk3 | -0,56 | 2,0E-02 |
| Cd101 | -0,61 | 2,0E-02 |
| Ergic1 | 0,81 | 2,0E-02 |
| Birc5 | -1,90 | 2,0E-02 |
| Sorbs3 | -0,92 | 2,0E-02 |
| Fam13b | -0,73 | 2,0E-02 |
| Hspa13 | 0,72 | 2,0E-02 |
| H2-DMb2 | -0,62 | 2,0E-02 |
| F2rl2 | -1,21 | 2,0E-02 |
| Atpaf1 | 0,54 | 2,0E-02 |
| Slmap | 0,92 | 2,0E-02 |
| Cbfa2t2 | 0,68 | 2,0E-02 |
| Cdc123 | 0,54 | 2,0E-02 |
| Lcorl | 1,45 | 2,0E-02 |
| Rhbg; Tsacc | -0,61 | 2,0E-02 |
| B3gnt5 | -1,21 | 2,1E-02 |
| B4galt1 | 0,65 | 2,1E-02 |
| Uhrf1bp1 | 0,73 | 2,1E-02 |
| Fxr1 | 0,74 | 2,1E-02 |
| Palld | 0,84 | 2,1E-02 |
| Kpna2 | -0,86 | 2,1E-02 |
| Pigl | 1,01 | 2,1E-02 |
| Klra3 | -1,22 | 2,1E-02 |
| Ip6k2 | 0,72 | 2,1E-02 |
| Lrrc8d | 0,90 | 2,1E-02 |
| Def8 | 0,68 | 2,1E-02 |
| Snx2 | 0,54 | 2,1E-02 |
| Rdh12 | -0,75 | 2,1E-02 |
| Rpl22l1 | -0,94 | 2,1E-02 |
| Hmg20a | 0,67 | 2,1E-02 |
| Car5b | 1,19 | 2,1E-02 |
| Fam196b | -0,97 | 2,1E-02 |
| Itpr3; Mir7677 | 0,83 | 2,1E-02 |
| Fam122b | 0,99 | 2,1E-02 |
| Samd8 | 1,25 | 2,2E-02 |
| Primpol | 0,78 | 2,2E-02 |
| Mark4 | 1,00 | 2,2E-02 |
| Setmar | -0,61 | 2,2E-02 |
| Kctd13 | 0,82 | 2,2E-02 |
| Hmga1; Hmga1-rs1 | 0,77 | 2,2E-02 |
| B4galt5 | -1,20 | 2,2E-02 |
| Nrg1 | -0,76 | 2,2E-02 |
| Ms4a4c | -0,80 | 2,2E-02 |
| Odc1 | 0,71 | 2,2E-02 |
| Slco3a1 | -0,70 | 2,2E-02 |
| Fpgt | 0,78 | 2,2E-02 |
| Mms22l | -0,70 | 2,2E-02 |
| Ikzf1 | -0,58 | 2,2E-02 |
| St3gal4 | -0,73 | 2,2E-02 |
| Tle6 | 0,84 | 2,2E-02 |
| Chpf2; Mir671 | 0,99 | 2,2E-02 |
| Tmem230 | 0,64 | 2,2E-02 |
| Paxbp1 | 1,03 | 2,2E-02 |
| Top2a | -1,23 | 2,2E-02 |
| Chst2 | -0,64 | 2,2E-02 |
| Zdhhc6 | 0,68 | 2,2E-02 |
| Tmed4 | 0,79 | 2,2E-02 |
| Zfp36 | -0,57 | 2,2E-02 |
| Rabl2 | -0,87 | 2,2E-02 |
| Poli | 0,62 | 2,2E-02 |
| Tax1bp1 | 0,90 | 2,2E-02 |
| Enpp4 | -0,93 | 2,2E-02 |
| Bre | 1,04 | 2,2E-02 |
| Snx24 | 0,81 | 2,2E-02 |
| Ankzf1 | 0,73 | 2,3E-02 |
| Gcnt2 | -1,26 | 2,3E-02 |
| Cyp39a1 | -1,07 | 2,3E-02 |
| Carf | 0,64 | 2,3E-02 |
| Gna15 | -1,56 | 2,3E-02 |
| Bicc1 | -0,82 | 2,3E-02 |
| Man2a1 | 0,80 | 2,3E-02 |
| Ruvbl1 | -0,68 | 2,3E-02 |
| Trp53i11 | -0,62 | 2,3E-02 |
| Traf6 | 0,80 | 2,3E-02 |
| Ubxn2a | 0,56 | 2,3E-02 |
| Cldnd1 | 0,98 | 2,3E-02 |
| Gfm2 | 0,60 | 2,3E-02 |
| Lonrf1 | 0,61 | 2,3E-02 |
| Nr1h3 | -1,08 | 2,3E-02 |
| Pdf; Cog8 | -0,71 | 2,4E-02 |
| Cyp51 | -0,92 | 2,4E-02 |
| Atrnl1 | -0,78 | 2,4E-02 |
| Grhpr | 1,23 | 2,4E-02 |
| Polq | -0,77 | 2,4E-02 |
| Zfp667 | -0,92 | 2,4E-02 |
| Tead3 | -0,56 | 2,4E-02 |
| Rbm12; Cpne1 | 0,69 | 2,4E-02 |
| Nprl3 | 0,71 | 2,4E-02 |
| Nat9 | 1,67 | 2,4E-02 |
| Aunip | -1,19 | 2,4E-02 |
| Riok3 | 0,72 | 2,4E-02 |
| Dera | 0,81 | 2,4E-02 |
| Abca9 | -1,89 | 2,4E-02 |
| Asrgl1 | 0,70 | 2,4E-02 |
| S100a4 | -1,00 | 2,4E-02 |
| Ppm1a | 0,63 | 2,4E-02 |
| Gpatch4 | -0,91 | 2,4E-02 |
| Cenpi | -0,68 | 2,4E-02 |
| Rab18 | 0,62 | 2,4E-02 |
| Acsf2 | 0,63 | 2,4E-02 |
| Cdc20 | -1,24 | 2,4E-02 |
| Defb46 | -0,66 | 2,4E-02 |
| Gp1bb; Sept5 | -0,85 | 2,4E-02 |
| Slc2a9 | 0,83 | 2,4E-02 |
| Papola | 0,68 | 2,4E-02 |
| Adi1 | 0,59 | 2,4E-02 |
| Mast3 | 0,71 | 2,4E-02 |
| Grk5 | 0,89 | 2,4E-02 |
| Dgkh | -0,66 | 2,4E-02 |
| Fbxl4 | 0,55 | 2,4E-02 |
| Dars | 0,73 | 2,4E-02 |
| Myoz1 | 0,58 | 2,5E-02 |
| Id1 | -0,86 | 2,5E-02 |
| Ltbp3 | 0,74 | 2,5E-02 |
| Rngtt | 0,74 | 2,5E-02 |
| Msrb2 | -1,13 | 2,5E-02 |
| Strbp | -0,59 | 2,5E-02 |
| Nmt2 | 0,78 | 2,5E-02 |
| Phldb2 | -0,83 | 2,5E-02 |
| Vipas39 | 0,74 | 2,5E-02 |
| Loxl3 | 0,73 | 2,5E-02 |
| Acadl | 0,82 | 2,5E-02 |
| Ankrd17 | 0,81 | 2,5E-02 |
| Rab4a | 1,29 | 2,5E-02 |
| Lgals3bp | 0,90 | 2,5E-02 |
| Cryzl1 | 0,82 | 2,5E-02 |
| Ly6e | -1,44 | 2,5E-02 |
| Bgn | -0,86 | 2,5E-02 |
| Mecom | -0,52 | 2,5E-02 |
| Maged2 | -0,60 | 2,5E-02 |
| Ogt | 0,60 | 2,5E-02 |
| B4galnt1 | -0,97 | 2,6E-02 |
| Ear10 | -1,36 | 2,6E-02 |
| Cab39 | 0,54 | 2,6E-02 |
| Wee1 | 0,53 | 2,6E-02 |
| Pkmyt1 | -0,81 | 2,6E-02 |
| Olfr1299; Olfr1300-ps1; Olfr1295 | -0,75 | 2,6E-02 |
| Cnn3 | -0,83 | 2,6E-02 |
| Efcab14 | 0,74 | 2,6E-02 |
| Bod1l | 0,80 | 2,6E-02 |
| Mamdc2 | -2,59 | 2,6E-02 |
| Klf3 | 0,78 | 2,6E-02 |
| Pgs1 | -0,66 | 2,6E-02 |
| Pfkp | 1,46 | 2,6E-02 |
| Ddx46 | -0,81 | 2,6E-02 |
| Asb7 | 0,57 | 2,6E-02 |
| Igfbp5 | -1,35 | 2,6E-02 |
| Sp140 | 0,94 | 2,6E-02 |
| Rnf125 | -0,67 | 2,6E-02 |
| Nfam1 | -1,23 | 2,6E-02 |
| Sos2 | 0,68 | 2,6E-02 |
| Opa1 | 0,91 | 2,6E-02 |
| Rgl2 | 1,22 | 2,6E-02 |
| Ralgapa2 | 0,79 | 2,6E-02 |
| Tpi1 | 0,90 | 2,6E-02 |
| Arhgef3 | 1,01 | 2,6E-02 |
| Tgm1 | 0,67 | 2,6E-02 |
| Gpbp1 | 0,66 | 2,6E-02 |
| Shcbp1 | -0,80 | 2,6E-02 |
| Irf2bpl | 0,91 | 2,6E-02 |
| Stac2 | -0,93 | 2,6E-02 |
| Chst14 | 0,55 | 2,6E-02 |
| Mblac1 | -0,57 | 2,6E-02 |
| Smc2 | -0,79 | 2,6E-02 |
| Necap1 | 0,66 | 2,6E-02 |
| Lamtor4 | -0,54 | 2,6E-02 |
| Gtpbp10 | 0,86 | 2,7E-02 |
| Malt1 | -0,97 | 2,7E-02 |
| Tmem106c | 0,75 | 2,7E-02 |
| Tpm2 | -0,87 | 2,7E-02 |
| Srpx2 | -1,16 | 2,7E-02 |
| 4930438A08Rik | -0,71 | 2,7E-02 |
| Gm15446 | 0,88 | 2,7E-02 |
| Creb3l2 | 1,26 | 2,7E-02 |
| Gprasp1; Armcx5 | 0,76 | 2,7E-02 |
| Gpr85 | -1,15 | 2,7E-02 |
| Gemin5 | 0,74 | 2,7E-02 |
| Pmvk | -0,86 | 2,7E-02 |
| Lage3 | 0,56 | 2,7E-02 |
| Spr | -0,77 | 2,7E-02 |
| Lpcat2 | -1,48 | 2,7E-02 |
| Clpb | 0,62 | 2,7E-02 |
| Hist1h3h | -0,92 | 2,7E-02 |
| Serpinb12 | -0,53 | 2,7E-02 |
| Nabp1 | 1,07 | 2,7E-02 |
| Cog7 | 0,68 | 2,7E-02 |
| Usp47 | 0,61 | 2,7E-02 |
| Ano10 | 1,03 | 2,7E-02 |
| Adam8 | 1,54 | 2,7E-02 |
| Manea | 0,81 | 2,7E-02 |
| 0610009O20Rik | -0,62 | 2,7E-02 |
| Hist1h2ao; Hist1h2ap; Hist1h2ai; Hist1h2ah | -0,72 | 2,7E-02 |
| Hist1h2ap | -0,72 | 2,7E-02 |
| Siah1b | -0,52 | 2,7E-02 |
| Fam234a | 0,96 | 2,8E-02 |
| Lsm2 | -0,93 | 2,8E-02 |
| 1700003E24Rik; BC061195 | -0,99 | 2,8E-02 |
| Crls1 | 0,52 | 2,8E-02 |
| Tatdn2 | -0,84 | 2,8E-02 |
| Pot1b | -0,76 | 2,8E-02 |
| Slc38a9 | 0,71 | 2,8E-02 |
| Rasal3 | -1,08 | 2,8E-02 |
| Ptger4 | -0,68 | 2,8E-02 |
| Zfp608 | -0,70 | 2,8E-02 |
| Bmp1 | -0,82 | 2,8E-02 |
| Kif9 | 0,56 | 2,8E-02 |
| Lamc1 | 1,01 | 2,8E-02 |
| Egln1 | 0,88 | 2,8E-02 |
| Cd81 | 0,68 | 2,8E-02 |
| Gm10134 | 0,96 | 2,8E-02 |
| Ola1 | 0,59 | 2,8E-02 |
| Dcxr | 0,70 | 2,8E-02 |
| Yeats4 | -0,73 | 2,8E-02 |
| Pgm2 | 0,90 | 2,8E-02 |
| Uba3 | 0,61 | 2,8E-02 |
| 2900060B14Rik | -1,02 | 2,8E-02 |
| Cxcr4 | -0,82 | 2,8E-02 |
| Tns1 | -0,81 | 2,8E-02 |
| Smc6 | -0,58 | 2,8E-02 |
| Crip1 | -0,91 | 2,8E-02 |
| Shisa9 | -0,57 | 2,8E-02 |
| Hist1h2ad; Hist1h3b; Hist1h3d | -0,57 | 2,8E-02 |
| Tmem242 | 0,91 | 2,9E-02 |
| Slc24a5 | -0,71 | 2,9E-02 |
| 1110007C09Rik | 0,98 | 2,9E-02 |
| Kdm5b | 0,59 | 2,9E-02 |
| Hyal1; Nat6; Hyal3 | 0,83 | 2,9E-02 |
| Tex30 | -0,57 | 2,9E-02 |
| Nlgn2 | 0,95 | 2,9E-02 |
| Gramd1a | 0,66 | 2,9E-02 |
| A530064D06Rik | 0,86 | 2,9E-02 |
| Ear7; Ear6 | -1,14 | 2,9E-02 |
| Thnsl1 | 0,59 | 2,9E-02 |
| Stoml2 | 0,93 | 2,9E-02 |
| Gypc | 0,72 | 2,9E-02 |
| Brat1 | 0,61 | 2,9E-02 |
| Llgl2 | -0,54 | 2,9E-02 |
| Alg2 | 0,64 | 2,9E-02 |
| Pemt | -0,81 | 2,9E-02 |
| Gatsl3 | 1,18 | 2,9E-02 |
| Cgnl1 | 0,79 | 2,9E-02 |
| Gramd3 | 1,04 | 2,9E-02 |
| Selp | -2,18 | 2,9E-02 |
| Ppp2r3d | -1,19 | 2,9E-02 |
| Crybb3 | -0,49 | 2,9E-02 |
| Cbr4 | 0,61 | 2,9E-02 |
| Gtf2a1 | 0,58 | 3,0E-02 |
| Hist1h2af | -0,64 | 3,0E-02 |
| Glg1 | 0,60 | 3,0E-02 |
| Slc25a1 | 0,57 | 3,0E-02 |
| 4930402H24Rik; Gm14057 | -1,08 | 3,0E-02 |
| Dbp | 1,23 | 3,0E-02 |
| Zdhhc1 | 0,63 | 3,0E-02 |
| Ppp1r7 | 0,87 | 3,0E-02 |
| Hpn | -0,60 | 3,0E-02 |
| Dnajb8 | -0,63 | 3,0E-02 |
| Tiprl | 0,77 | 3,0E-02 |
| Maoa | 1,29 | 3,0E-02 |
| Mapk9 | 0,59 | 3,0E-02 |
| Gm14190 | -0,67 | 3,0E-02 |
| Cnn2 | -0,89 | 3,0E-02 |
| Ccdc152 | -0,69 | 3,0E-02 |
| Ern1 | 1,15 | 3,0E-02 |
| Atf7ip | 0,93 | 3,0E-02 |
| Acy3 | 1,00 | 3,0E-02 |
| Yme1l1 | 0,60 | 3,0E-02 |
| Cln8 | 1,12 | 3,0E-02 |
| Tecpr2 | 0,59 | 3,0E-02 |
| Mrpl17 | 0,70 | 3,0E-02 |
| Gar1 | -0,64 | 3,0E-02 |
| Zfp738 | 1,10 | 3,1E-02 |
| Stxbp5 | 0,84 | 3,1E-02 |
| Ube3a | 0,62 | 3,1E-02 |
| Spty2d1 | 0,74 | 3,1E-02 |
| Umps | -0,58 | 3,1E-02 |
| Sacm1l | 0,75 | 3,1E-02 |
| Cd40 | 0,58 | 3,1E-02 |
| Man1a | 0,76 | 3,1E-02 |
| Bckdhb | 0,73 | 3,1E-02 |
| Zfp932 | 0,98 | 3,1E-02 |
| Hsdl2 | 0,73 | 3,1E-02 |
| Lrrc41 | 0,63 | 3,1E-02 |
| Trim44 | 0,79 | 3,1E-02 |
| Ear12; Ear2; Ear3 | -1,66 | 3,1E-02 |
| March5 | 0,49 | 3,1E-02 |
| Smg1 | 0,49 | 3,1E-02 |
| Timm17a | 0,52 | 3,1E-02 |
| Scpep1 | 0,62 | 3,1E-02 |
| Dscr3 | 0,72 | 3,1E-02 |
| Mccc1 | 0,75 | 3,1E-02 |
| Ghitm | 0,82 | 3,1E-02 |
| Cdk20 | 1,37 | 3,1E-02 |
| Slamf6 | -0,80 | 3,1E-02 |
| Gm16253 | -0,52 | 3,1E-02 |
| Slc14a1 | -1,17 | 3,1E-02 |
| Orai3 | -0,76 | 3,1E-02 |
| Lbh | 0,51 | 3,1E-02 |
| Naaa | -0,84 | 3,1E-02 |
| 1810013L24Rik | 0,49 | 3,1E-02 |
| Tnnc1 | 0,76 | 3,1E-02 |
| Siah2 | 0,85 | 3,1E-02 |
| Sil1 | 0,85 | 3,1E-02 |
| Amica1 | -1,12 | 3,2E-02 |
| Arcn1 | 0,76 | 3,2E-02 |
| Lrba | 0,91 | 3,2E-02 |
| Cep170 | 0,63 | 3,2E-02 |
| 4930430F08Rik | 0,70 | 3,2E-02 |
| Mthfd1l | 0,59 | 3,2E-02 |
| Abracl | -0,60 | 3,2E-02 |
| Zfp142 | -0,64 | 3,2E-02 |
| Iqcf3 | -0,63 | 3,2E-02 |
| Gstm5 | 0,87 | 3,2E-02 |
| Arg1 | 1,69 | 3,2E-02 |
| Srrt | -0,73 | 3,2E-02 |
| Kif11 | -0,63 | 3,2E-02 |
| Hectd1 | 0,64 | 3,2E-02 |
| Arfip1 | 0,62 | 3,2E-02 |
| Plk1 | -2,09 | 3,2E-02 |
| Spc24 | -0,88 | 3,2E-02 |
| Gm14326 | 0,60 | 3,2E-02 |
| Bloc1s6 | 0,60 | 3,2E-02 |
| 4930503B20Rik | -0,88 | 3,2E-02 |
| Slc25a13 | 0,60 | 3,3E-02 |
| Rnf10 | 1,02 | 3,3E-02 |
| Maf1 | -0,56 | 3,3E-02 |
| Agpat3 | 0,89 | 3,3E-02 |
| Fam210a; Mir7219 | 0,81 | 3,3E-02 |
| Psmd4 | 0,79 | 3,3E-02 |
| Gm4583 | -0,50 | 3,3E-02 |
| Asf1b | -1,75 | 3,3E-02 |
| Stag1 | -0,53 | 3,3E-02 |
| Gm6682 | -0,74 | 3,3E-02 |
| Srrm2 | 0,55 | 3,3E-02 |
| 2810417H13Rik | -2,09 | 3,3E-02 |
| Chil1 | -0,67 | 3,3E-02 |
| Sgol1 | -1,09 | 3,3E-02 |
| Pomt2 | 0,60 | 3,3E-02 |
| Pfkfb3 | 0,60 | 3,3E-02 |
| Zdhhc5 | 0,73 | 3,3E-02 |
| Ankmy2 | 0,54 | 3,4E-02 |
| Uso1 | 0,58 | 3,4E-02 |
| Dchs1 | -0,86 | 3,4E-02 |
| Pgm3 | 0,51 | 3,4E-02 |
| Atg4c | 0,72 | 3,4E-02 |
| Slx4 | 0,56 | 3,4E-02 |
| Cdk8 | 0,60 | 3,4E-02 |
| Ganab | 1,10 | 3,4E-02 |
| Fgd6 | 0,95 | 3,4E-02 |
| Ociad2 | 0,74 | 3,4E-02 |
| Rnf123 | 0,61 | 3,4E-02 |
| Hook1 | -0,51 | 3,4E-02 |
| Lifr | -0,70 | 3,4E-02 |
| Cnot2 | 0,50 | 3,4E-02 |
| Kif20b | -1,39 | 3,4E-02 |
| Cd93 | 0,69 | 3,4E-02 |
| Slc38a10 | 1,30 | 3,4E-02 |
| Abtb1 | 0,59 | 3,4E-02 |
| Dtymk | -0,59 | 3,4E-02 |
| Kti12 | -0,80 | 3,4E-02 |
| Vezt; Mir331 | 0,81 | 3,5E-02 |
| Emc8 | -0,69 | 3,5E-02 |
| Hells | -0,69 | 3,5E-02 |
| Slc26a2 | -0,58 | 3,5E-02 |
| Insig2 | 0,62 | 3,5E-02 |
| Ksr2 | -1,65 | 3,5E-02 |
| Aurkb | -0,82 | 3,5E-02 |
| Mettl9 | 0,79 | 3,5E-02 |
| Mapkapk2 | -0,58 | 3,5E-02 |
| Spcs3 | 0,70 | 3,5E-02 |
| Adamts7 | -0,48 | 3,5E-02 |
| Phf10 | 0,61 | 3,5E-02 |
| Dhx30 | 0,88 | 3,5E-02 |
| Dtwd1 | 0,51 | 3,5E-02 |
| Cuta | -0,53 | 3,5E-02 |
| Prkci | 0,88 | 3,5E-02 |
| Bex1 | -0,85 | 3,5E-02 |
| Usp39 | 0,55 | 3,5E-02 |
| Nop9 | 0,55 | 3,5E-02 |
| Jmjd4 | -0,82 | 3,5E-02 |
| Gm14325 | 0,65 | 3,5E-02 |
| Cntln | -0,55 | 3,5E-02 |
| Slc35a2 | 0,71 | 3,5E-02 |
| Git1 | 0,83 | 3,5E-02 |
| Neto2 | -0,58 | 3,5E-02 |
| Adck2 | 0,62 | 3,5E-02 |
| Olfr845 | -0,53 | 3,5E-02 |
| Crisp3 | -0,54 | 3,6E-02 |
| Hmgn3 | -1,22 | 3,6E-02 |
| Ppdpf | 0,83 | 3,6E-02 |
| Mgst1 | 0,56 | 3,6E-02 |
| Sdccag8 | 0,57 | 3,6E-02 |
| Plin2 | 0,62 | 3,6E-02 |
| Calcrl | 1,05 | 3,6E-02 |
| Epha7 | -0,48 | 3,6E-02 |
| Stmn1 | -1,40 | 3,6E-02 |
| Akirin1 | 0,54 | 3,6E-02 |
| Med23 | 0,56 | 3,6E-02 |
| Sqstm1 | 0,70 | 3,6E-02 |
| 9930021J03Rik | 0,92 | 3,6E-02 |
| Ubr2 | 0,90 | 3,6E-02 |
| Nck2 | 0,89 | 3,6E-02 |
| Adam9 | 0,73 | 3,6E-02 |
| Gpr34 | -0,96 | 3,6E-02 |
| Pdha1 | 0,51 | 3,6E-02 |
| Prg3 | -1,05 | 3,6E-02 |
| Foxm1; 4933413G19Rik | -2,57 | 3,6E-02 |
| Stard9 | -0,65 | 3,6E-02 |
| BC026585 | 0,55 | 3,6E-02 |
| Pdlim1 | -1,01 | 3,6E-02 |
| Pld1 | 0,93 | 3,6E-02 |
| Coro7 | 0,76 | 3,6E-02 |
| Zfp938 | 0,91 | 3,6E-02 |
| Zmat1 | 0,97 | 3,6E-02 |
| Smarce1 | 0,58 | 3,7E-02 |
| Mmp9 | 0,87 | 3,7E-02 |
| Galnt9 | -1,48 | 3,7E-02 |
| Rab19 | -1,05 | 3,7E-02 |
| Plod1 | 0,98 | 3,7E-02 |
| Reps2 | 0,63 | 3,7E-02 |
| Ireb2 | 0,56 | 3,7E-02 |
| Prkaa1 | 0,64 | 3,7E-02 |
| Frat2 | -0,57 | 3,7E-02 |
| Gpatch8 | 0,82 | 3,7E-02 |
| Hist1h3g | -0,55 | 3,7E-02 |
| Vps13d | 1,02 | 3,7E-02 |
| Cog1 | -0,54 | 3,7E-02 |
| Ube3b | 0,83 | 3,7E-02 |
| Limd2 | -0,72 | 3,7E-02 |
| Bub1b | -0,79 | 3,7E-02 |
| Papd7 | -0,68 | 3,7E-02 |
| Naa25 | 0,59 | 3,7E-02 |
| Gm11564 | -0,51 | 3,7E-02 |
| 6030408B16Rik | -0,50 | 3,7E-02 |
| Zak | 1,07 | 3,7E-02 |
| Samd15 | -0,51 | 3,7E-02 |
| Hspb11 | 1,22 | 3,7E-02 |
| 1600012H06Rik | 0,73 | 3,7E-02 |
| Gtf3c1 | 0,68 | 3,8E-02 |
| Lrrc28 | 0,61 | 3,8E-02 |
| Ddx42 | 0,78 | 3,8E-02 |
| Zyg11b | 0,78 | 3,8E-02 |
| Zdhhc21 | 0,65 | 3,8E-02 |
| Arih1 | 0,57 | 3,8E-02 |
| Cphx2 | -0,61 | 3,8E-02 |
| Vps8 | 1,25 | 3,8E-02 |
| Pyhin1 | 0,79 | 3,8E-02 |
| Tbcc | -0,56 | 3,8E-02 |
| Vmn2r49 | 0,53 | 3,8E-02 |
| Phf20 | 0,55 | 3,8E-02 |
| Lmo7 | -1,17 | 3,8E-02 |
| Aurka | -0,52 | 3,8E-02 |
| Lyl1 | -0,78 | 3,8E-02 |
| Fbxo44 | 0,56 | 3,8E-02 |
| Sertad2 | 0,90 | 3,8E-02 |
| Ppip5k2 | -0,52 | 3,8E-02 |
| Rnf169 | 0,64 | 3,8E-02 |
| Prrg4 | -0,55 | 3,8E-02 |
| Il6ra | -0,96 | 3,8E-02 |
| Fut7 | -0,70 | 3,8E-02 |
| Fam136a | 0,64 | 3,8E-02 |
| Siae | 0,89 | 3,8E-02 |
| Hs6st1 | 0,48 | 3,8E-02 |
| Gm14399 | 0,62 | 3,8E-02 |
| Flot1 | 0,67 | 3,8E-02 |
| Cd274 | 1,23 | 3,8E-02 |
| Ccng1 | 0,65 | 3,9E-02 |
| Galm | 0,78 | 3,9E-02 |
| Larp7 | -0,77 | 3,9E-02 |
| Mettl15 | 0,72 | 3,9E-02 |
| Skap2 | -0,66 | 3,9E-02 |
| Rnf217 | 0,49 | 3,9E-02 |
| Cbx7 | 0,47 | 3,9E-02 |
| Aggf1 | -0,73 | 3,9E-02 |
| Ammecr1l | 0,57 | 3,9E-02 |
| Olfr828 | -0,70 | 3,9E-02 |
| Pelp1 | -0,69 | 3,9E-02 |
| Hdhd2 | 0,51 | 3,9E-02 |
| Gp1ba | -0,89 | 3,9E-02 |
| Mocs2 | 0,59 | 3,9E-02 |
| Ralgapa1 | 0,62 | 3,9E-02 |
| Thsd7a | -0,51 | 3,9E-02 |
| Trappc9 | 0,55 | 3,9E-02 |
| Sufu | 0,74 | 3,9E-02 |
| Avl9 | 0,78 | 3,9E-02 |
| Pmf1 | -1,15 | 3,9E-02 |
| Pafah1b2 | 0,61 | 3,9E-02 |
| Dqx1 | 0,67 | 3,9E-02 |
| Skint10 | -0,55 | 4,0E-02 |
| Nr4a2 | -0,96 | 4,0E-02 |
| Xkr8 | 0,60 | 4,0E-02 |
| Prkaa2 | 0,82 | 4,0E-02 |
| Gm14431; Gm8898 | 0,81 | 4,0E-02 |
| Mmp27 | 1,30 | 4,0E-02 |
| Mdga1 | -0,56 | 4,0E-02 |
| Mkrn2 | 0,80 | 4,0E-02 |
| Armc9 | 0,56 | 4,0E-02 |
| Ninj1 | 0,53 | 4,0E-02 |
| Arl4c | -0,62 | 4,0E-02 |
| Casp7 | 0,83 | 4,0E-02 |
| Alg12 | 0,58 | 4,0E-02 |
| Trmt5 | 0,55 | 4,0E-02 |
| Kctd17 | 0,55 | 4,0E-02 |
| Rab40c | 0,78 | 4,0E-02 |
| Lcmt2 | -0,53 | 4,0E-02 |
| Gfi1b | -0,67 | 4,0E-02 |
| Kdm3a | 0,71 | 4,0E-02 |
| Tgfbr1 | -1,35 | 4,0E-02 |
| Atg2a | 0,78 | 4,0E-02 |
| Fads3 | -0,55 | 4,0E-02 |
| Phka2 | 0,93 | 4,0E-02 |
| Srpk1 | 0,59 | 4,0E-02 |
| Hnrnpa1; Gm10052 | -0,50 | 4,0E-02 |
| Mtmr9 | 0,61 | 4,0E-02 |
| Fgl2 | 0,73 | 4,0E-02 |
| Ptar1 | 0,63 | 4,1E-02 |
| Gatm | 0,86 | 4,1E-02 |
| Steap1 | -1,16 | 4,1E-02 |
| Gm13305; Il11ra2; Gm2002 | -0,91 | 4,1E-02 |
| Rabggtb; Snord45c | 0,69 | 4,1E-02 |
| Madd | -0,68 | 4,1E-02 |
| Evl | -0,60 | 4,1E-02 |
| Gm10424 | -0,84 | 4,1E-02 |
| Tfpi | 0,92 | 4,1E-02 |
| Hmgb1 | -0,52 | 4,1E-02 |
| Dclre1a | 0,50 | 4,1E-02 |
| Psg27 | -0,70 | 4,1E-02 |
| Ptprv | 0,87 | 4,1E-02 |
| AI606181 | 0,59 | 4,1E-02 |
| Itgal | -1,25 | 4,1E-02 |
| Aga | 0,56 | 4,1E-02 |
| Olfr411 | -0,66 | 4,1E-02 |
| Cyp20a1 | -0,50 | 4,1E-02 |
| Pcgf2 | 0,88 | 4,1E-02 |
| Eid1 | 0,57 | 4,1E-02 |
| Pdik1l | 0,53 | 4,1E-02 |
| Zkscan3 | 0,56 | 4,1E-02 |
| Il12rb2 | -0,96 | 4,1E-02 |
| Far2 | -0,49 | 4,1E-02 |
| Gm8096 | 0,48 | 4,1E-02 |
| Hist1h2ai | -0,61 | 4,1E-02 |
| Camk1d | -0,82 | 4,1E-02 |
| Taar7d | -0,69 | 4,2E-02 |
| Zfp787 | 1,04 | 4,2E-02 |
| Egr2 | -0,87 | 4,2E-02 |
| Eea1 | 0,69 | 4,2E-02 |
| Gm17268 | -0,63 | 4,2E-02 |
| Stxbp1 | 0,58 | 4,2E-02 |
| Wdr13 | -0,73 | 4,2E-02 |
| Psd3 | 0,87 | 4,2E-02 |
| Nt5c | -1,02 | 4,2E-02 |
| Sdf2l1 | -0,58 | 4,2E-02 |
| Panx1 | 0,78 | 4,2E-02 |
| Pdia6 | 0,92 | 4,2E-02 |
| Eif4e2 | 0,47 | 4,2E-02 |
| Tmem232 | -0,66 | 4,2E-02 |
| Cerkl; Neurod1 | 0,65 | 4,2E-02 |
| Elmod2 | 0,71 | 4,2E-02 |
| Mpc1 | 0,63 | 4,2E-02 |
| Tnfrsf12a | -0,58 | 4,2E-02 |
| Zfp397 | 0,52 | 4,2E-02 |
| Usb1 | 0,68 | 4,2E-02 |
| Krtcap2 | 0,73 | 4,2E-02 |
| Acss1 | 1,41 | 4,2E-02 |
| Slc37a4 | 0,86 | 4,2E-02 |
| Rasl10a | 0,55 | 4,2E-02 |
| Ube2c | -1,57 | 4,2E-02 |
| Rrad | -0,79 | 4,2E-02 |
| Vps39 | 0,73 | 4,2E-02 |
| Mlana | -0,48 | 4,2E-02 |
| Slc2a6 | -0,67 | 4,3E-02 |
| Gab1 | 0,68 | 4,3E-02 |
| Gab2 | 0,63 | 4,3E-02 |
| Gpr108 | 0,56 | 4,3E-02 |
| Car9 | 0,56 | 4,3E-02 |
| Marf1 | 0,74 | 4,3E-02 |
| Nars2 | 0,55 | 4,3E-02 |
| Xlr4a | -0,48 | 4,3E-02 |
| Tfcp2 | 0,66 | 4,3E-02 |
| Kdm6b | 1,52 | 4,3E-02 |
| Zfand1 | 0,47 | 4,3E-02 |
| Sec13 | 0,58 | 4,3E-02 |
| Chd1 | 0,57 | 4,3E-02 |
| Gm14403 | 0,79 | 4,3E-02 |
| Nxt2 | 0,51 | 4,3E-02 |
| Utp11l | 0,46 | 4,3E-02 |
| Ppp3r1 | 0,67 | 4,3E-02 |
| Gm10382; Gm19631 | 1,75 | 4,3E-02 |
| Plekha8 | 1,12 | 4,3E-02 |
| Pi4k2b | 0,52 | 4,3E-02 |
| Ncoa1 | 0,93 | 4,3E-02 |
| Tbc1d12 | 0,48 | 4,3E-02 |
| Fam83f | -0,47 | 4,3E-02 |
| Rev3l | 0,71 | 4,3E-02 |
| Olfr311 | -0,59 | 4,3E-02 |
| Gm8356 | -0,49 | 4,3E-02 |
| Gstz1 | 0,88 | 4,4E-02 |
| Gdpd3 | 0,68 | 4,4E-02 |
| Caml | 0,56 | 4,4E-02 |
| Cers5 | -0,79 | 4,4E-02 |
| Zfp873 | 0,72 | 4,4E-02 |
| Ugdh | 0,71 | 4,4E-02 |
| Utp14b; Acsl3 | -1,49 | 4,4E-02 |
| Dnajc13 | 0,53 | 4,4E-02 |
| Pbk | -2,79 | 4,4E-02 |
| Ifih1 | 0,70 | 4,4E-02 |
| Cib2 | -1,26 | 4,4E-02 |
| Dkc1 | -0,96 | 4,4E-02 |
| Tnfrsf1b | -0,78 | 4,4E-02 |
| Prkag1 | -0,64 | 4,4E-02 |
| Nme1 | -0,65 | 4,4E-02 |
| Tmem132a | -0,78 | 4,4E-02 |
| Hist1h2ah | -0,72 | 4,4E-02 |
| Cadm1 | -0,59 | 4,4E-02 |
| Trmt61a | -0,60 | 4,4E-02 |
| Fam175b | 0,58 | 4,4E-02 |
| Slc39a4 | -0,71 | 4,4E-02 |
| Uba5 | 0,63 | 4,4E-02 |
| Etohi1 | 0,54 | 4,4E-02 |
| Klhl6 | -1,46 | 4,4E-02 |
| Il34 | -0,47 | 4,4E-02 |
| Usp40 | 0,55 | 4,4E-02 |
| Ech1 | 0,51 | 4,4E-02 |
| 3110009E18Rik | -0,92 | 4,4E-02 |
| Mtmr2 | 0,49 | 4,4E-02 |
| 4930522L14Rik | 0,73 | 4,5E-02 |
| Nbr1 | 0,96 | 4,5E-02 |
| Ap3b1 | 0,53 | 4,5E-02 |
| Cacfd1 | 0,63 | 4,5E-02 |
| St3gal1 | -0,48 | 4,5E-02 |
| Ndfip1 | 0,45 | 4,5E-02 |
| Dnajc18 | 0,70 | 4,5E-02 |
| Ccnt1 | 0,61 | 4,5E-02 |
| Mtif2 | 0,67 | 4,5E-02 |
| Cnrip1 | -0,63 | 4,5E-02 |
| Tbx15 | -0,96 | 4,5E-02 |
| LOC100861916 | 0,59 | 4,5E-02 |
| Mkln1 | 0,63 | 4,5E-02 |
| Zfp317 | 0,69 | 4,5E-02 |
| Timeless | -1,06 | 4,5E-02 |
| H2-M3 | 0,67 | 4,5E-02 |
| Pde3b | 1,05 | 4,5E-02 |
| Coasy | 0,89 | 4,5E-02 |
| Gvin1 | 0,50 | 4,5E-02 |
| Sla | -1,21 | 4,5E-02 |
| Exo1 | -0,81 | 4,5E-02 |
| Akap11 | 0,55 | 4,5E-02 |
| 5430403G16Rik | 0,83 | 4,5E-02 |
| Serhl | 0,61 | 4,5E-02 |
| Msr1 | 0,63 | 4,5E-02 |
| Tmem194; 1700012D01Rik | 0,84 | 4,6E-02 |
| Tbl1x | 0,53 | 4,6E-02 |
| Tab3 | 0,62 | 4,6E-02 |
| Zfyve16 | 0,77 | 4,6E-02 |
| Gm10642 | -0,60 | 4,6E-02 |
| Gm14288; Gm14435 | 0,84 | 4,6E-02 |
| Gm14288; Gm14440 | 0,84 | 4,6E-02 |
| Ccdc6 | 0,70 | 4,6E-02 |
| Cth | 0,56 | 4,6E-02 |
| Kdm6a | 0,58 | 4,6E-02 |
| Fam160a1 | -0,56 | 4,6E-02 |
| Gm21814 | 0,49 | 4,6E-02 |
| Reps1 | 0,60 | 4,6E-02 |
| Arhgef25 | -0,52 | 4,6E-02 |
| Atp6v0b | -0,54 | 4,6E-02 |
| Atp5s | 1,15 | 4,6E-02 |
| Lsm10 | 0,56 | 4,6E-02 |
| Arid3b | 0,71 | 4,6E-02 |
| Ddx19b | 0,50 | 4,6E-02 |
| Fbxo3 | 0,76 | 4,6E-02 |
| Enkur | -0,71 | 4,6E-02 |
| Evi5 | 0,63 | 4,7E-02 |
| Notch1 | 0,52 | 4,7E-02 |
| Ccnb1 | -0,95 | 4,7E-02 |
| 5031414D18Rik | -0,77 | 4,7E-02 |
| Colgalt1 | -0,72 | 4,7E-02 |
| Gm14306 | 0,85 | 4,7E-02 |
| Mvd | -0,85 | 4,7E-02 |
| S100a1 | -0,73 | 4,7E-02 |
| Insig1 | -0,70 | 4,7E-02 |
| Fam216a | 0,48 | 4,7E-02 |
| Il11ra2 | -0,88 | 4,7E-02 |
| Slc38a2 | -0,83 | 4,7E-02 |
| Mief1 | 0,58 | 4,7E-02 |
| Sel1l | 0,57 | 4,7E-02 |
| Mtl5 | -0,52 | 4,7E-02 |
| Zbed3 | 0,62 | 4,7E-02 |
| Appl2 | 0,70 | 4,7E-02 |
| Shtn1 | -0,59 | 4,8E-02 |
| Gm12887 | -0,68 | 4,8E-02 |
| Trpv4 | 0,85 | 4,8E-02 |
| Mki67 | -1,48 | 4,8E-02 |
| Cyb561d2 | -0,59 | 4,8E-02 |
| Gen1 | -1,13 | 4,8E-02 |
| Vsig10l | -0,58 | 4,8E-02 |
| Dmxl1 | 0,50 | 4,8E-02 |
| Mrpl4 | 0,53 | 4,8E-02 |
| Klra17 | -0,57 | 4,8E-02 |
| Wdr48 | 0,46 | 4,8E-02 |
| Nrep | -0,48 | 4,8E-02 |
| Luc7l | 0,79 | 4,8E-02 |
| Cdca3 | -1,19 | 4,8E-02 |
| Crisp4 | 0,67 | 4,8E-02 |
| Slc10a7 | 0,52 | 4,8E-02 |
| Ly6d | -0,59 | 4,8E-02 |
| Cul3 | 0,44 | 4,8E-02 |
| Asph | 0,55 | 4,8E-02 |
| Jun | 0,51 | 4,8E-02 |
| Hist1h2bb | -0,64 | 4,8E-02 |
| Kctd12; Mir5130 | -1,80 | 4,8E-02 |
| Eps15 | 0,56 | 4,9E-02 |
| Naip2 | 0,83 | 4,9E-02 |
| Hist1h4f | -0,58 | 4,9E-02 |
| Cd320 | -0,52 | 4,9E-02 |
| Klhl21 | 0,72 | 4,9E-02 |
| Mb21d2 | -0,48 | 4,9E-02 |
| Fam107b | -0,50 | 4,9E-02 |
| Psmd5 | 0,54 | 4,9E-02 |
| Spire1 | 0,85 | 4,9E-02 |
| Sec16a | 0,59 | 4,9E-02 |
| Rnase6 | -0,78 | 4,9E-02 |
| Cltc | 1,15 | 4,9E-02 |
| Slc39a10 | -0,63 | 4,9E-02 |
| Mbnl3 | -0,91 | 4,9E-02 |
| Mcm7; Mir93; Mir25 | -1,34 | 4,9E-02 |
| Mapk1ip1 | 0,60 | 4,9E-02 |
| Vapb | 0,62 | 4,9E-02 |
| Lncpint | -0,75 | 4,9E-02 |
| Ap1ar | -0,53 | 4,9E-02 |
| Dnajc16 | 0,50 | 5,0E-02 |
| 1110004E09Rik | 0,65 | 5,0E-02 |
| Eef2k | 0,49 | 5,0E-02 |
| Rnf212 | -0,47 | 5,0E-02 |
| Surf1 | 0,88 | 5,0E-02 |
| Loxl1 | -1,00 | 5,0E-02 |
| Klrd1 | -1,25 | 5,0E-02 |
| Vdac1 | 0,61 | 5,0E-02 |
