# Supplementary material for: Metformin impacts the differentiation of mouse bone marrow cells into macrophages affecting tumour immunity
Source: Heliyon. 2024 Sep 11;10(18):e37792. doi: 10.1016/j.heliyon.2024.e37792 (PMC11417223; doi:10.1016/j.heliyon.2024.e37792)
Supplement: Multimedia component 2 [file mmc2.docx]

**Table S2. List of top gene ontology (GO) biological processes (BP) modulated in metformin-treated BMDMs.**

| **Description** | **GeneRatio** | **pvalue** | **p.adjust** |
| --- | --- | --- | --- |
| positive regulation of cytokine production | 61/1294 | 1,24E-12 | 6,91E-09 |
| cell chemotaxis | 45/1294 | 4,06E-12 | 1,02E-08 |
| regulation of cell-cell adhesion | 56/1294 | 8,76E-12 | 1,02E-08 |
| regulation of leukocyte proliferation | 40/1294 | 9,25E-12 | 1,02E-08 |
| regulation of lymphocyte proliferation | 38/1294 | 1,27E-11 | 1,02E-08 |
| mononuclear cell differentiation | 58/1294 | 1,41E-11 | 1,02E-08 |
| positive regulation of cell-cell adhesion | 42/1294 | 1,47E-11 | 1,02E-08 |
| leukocyte migration | 49/1294 | 1,47E-11 | 1,02E-08 |
| regulation of mononuclear cell proliferation | 38/1294 | 2,11E-11 | 1,31E-08 |
| regulation of vasculature development | 43/1294 | 2,90E-11 | 1,61E-08 |
| regulation of leukocyte cell-cell adhesion | 45/1294 | 3,84E-11 | 1,94E-08 |
| leukocyte cell-cell adhesion | 48/1294 | 4,92E-11 | 2,28E-08 |
| positive regulation of response to external stimulus | 52/1294 | 6,12E-11 | 2,62E-08 |
| regulation of angiogenesis | 42/1294 | 6,86E-11 | 2,73E-08 |
| regulation of T cell proliferation | 31/1294 | 1,54E-10 | 5,54E-08 |
| small GTPase mediated signal transduction | 51/1294 | 1,59E-10 | 5,54E-08 |
| positive regulation of leukocyte cell-cell adhesion | 36/1294 | 1,86E-10 | 6,09E-08 |
| positive regulation of cell adhesion | 54/1294 | 2,54E-10 | 7,88E-08 |
| T cell proliferation | 34/1294 | 2,97E-10 | 8,71E-08 |
| lymphocyte proliferation | 43/1294 | 3,34E-10 | 9,32E-08 |
| carboxylic acid biosynthetic process | 40/1294 | 4,19E-10 | 1,08E-07 |
| leukocyte proliferation | 45/1294 | 4,26E-10 | 1,08E-07 |
| organic acid biosynthetic process | 40/1294 | 4,64E-10 | 1,12E-07 |
| mononuclear cell proliferation | 43/1294 | 4,90E-10 | 1,14E-07 |
| myeloid leukocyte migration | 34/1294 | 5,45E-10 | 1,22E-07 |
| regulation of response to biotic stimulus | 43/1294 | 6,49E-10 | 1,39E-07 |
| regulation of T cell activation | 43/1294 | 7,81E-10 | 1,61E-07 |
| positive regulation of lymphocyte proliferation | 26/1294 | 9,52E-10 | 1,90E-07 |
| alcohol metabolic process | 43/1294 | 1,23E-09 | 2,33E-07 |
| positive regulation of mononuclear cell proliferation | 26/1294 | 1,30E-09 | 2,33E-07 |
| regulation of immune effector process | 49/1294 | 1,31E-09 | 2,33E-07 |
| temperature homeostasis | 30/1294 | 1,34E-09 | 2,33E-07 |
| regulation of leukocyte migration | 33/1294 | 1,50E-09 | 2,53E-07 |
| phospholipid metabolic process | 44/1294 | 1,61E-09 | 2,62E-07 |
| positive regulation of leukocyte proliferation | 27/1294 | 1,64E-09 | 2,62E-07 |
| regulation of leukocyte mediated immunity | 39/1294 | 1,76E-09 | 2,73E-07 |
| positive regulation of defense response | 37/1294 | 1,90E-09 | 2,87E-07 |
| regulation of protein catabolic process | 46/1294 | 2,01E-09 | 2,94E-07 |
| glycerolipid metabolic process | 44/1294 | 6,70E-09 | 9,57E-07 |
| leukocyte chemotaxis | 32/1294 | 7,97E-09 | 1,11E-06 |
| purine ribonucleotide metabolic process | 41/1294 | 1,66E-08 | 2,26E-06 |
| fatty acid biosynthetic process | 25/1294 | 1,90E-08 | 2,53E-06 |
| ribose phosphate metabolic process | 43/1294 | 2,29E-08 | 2,91E-06 |
| negative regulation of defense response | 32/1294 | 2,30E-08 | 2,91E-06 |
| ribonucleotide metabolic process | 42/1294 | 2,59E-08 | 3,21E-06 |
| proteasome-mediated ubiquitin-dependent protein catabolic process | 46/1294 | 2,75E-08 | 3,34E-06 |
| positive regulation of T cell proliferation | 20/1294 | 3,01E-08 | 3,53E-06 |
| regulation of inflammatory response | 40/1294 | 3,04E-08 | 3,53E-06 |
