# Supplementary material for: Metformin impacts the differentiation of mouse bone marrow cells into macrophages affecting tumour immunity
Source: Heliyon. 2024 Sep 11;10(18):e37792. doi: 10.1016/j.heliyon.2024.e37792 (PMC11417223; doi:10.1016/j.heliyon.2024.e37792)
Supplement: Multimedia component 3 [file mmc3.docx]

**Table S3. List of top down- (left) and up-regulated (right) gene ontology biological processes in metformin-treated BMDMs.**

| **NAME** | **NES** | **NOM p-val** | **NAME** | **NES** | **NOM p-val** |
| --- | --- | --- | --- | --- | --- |
| GOBP_MACROAUTOPHAGY | 2,2701013 | 0 | GOBP_LEUKOCYTE_TETHERING_OR_ROLLING | -2,3185 | 0 |
| GOBP_NADH_REGENERATION | 2,2526479 | 0 | GOBP_REGULATION_OF_LEUKOCYTE_TETHERING_OR_ROLLING | -2,2719092 | 0 |
| GOBP_GOLGI_VESICLE_TRANSPORT | 2,2289786 | 0 | GOBP_POSITIVE_REGULATION_OF_LEUKOCYTE_TETHERING_OR_ROLLING | -2,254877 | 0 |
| GOBP_GLYCOLYTIC_PROCESS_THROUGH_FRUCTOSE_6_PHOSPHATE | 2,2117527 | 0 | GOBP_POSITIVE_REGULATION_OF_LEUKOCYTE_ADHESION_TO_VASCULAR_ENDOTHELIAL_CELL | -2,2247605 | 0 |
| GOBP_RESPONSE_TO_TOPOLOGICALLY_INCORRECT_PROTEIN | 2,1953318 | 0 | GOBP_PEPTIDE_ANTIGEN_ASSEMBLY_WITH_MHC_PROTEIN_COMPLEX | -2,2051823 | 0 |
| GOBP_CELLULAR_RESPONSE_TO_TOPOLOGICALLY_INCORRECT_PROTEIN | 2,1937933 | 0 | GOBP_LEUKOCYTE_ADHESION_TO_VASCULAR_ENDOTHELIAL_CELL | -2,1350768 | 0 |
| GOBP_REGULATION_OF_MACROAUTOPHAGY | 2,1920106 | 0 | GOBP_REGULATION_OF_LEUKOCYTE_ADHESION_TO_VASCULAR_ENDOTHELIAL_CELL | -2,13044 | 0 |
| GOBP_GLUCOSE_CATABOLIC_PROCESS | 2,17957 | 0 | RENIN-ANGIOTENSIN SYSTEM | -2,1253343 | 0 |
| GOBP_MONOSACCHARIDE_CATABOLIC_PROCESS | 2,1738582 | 0 | STAPHYLOCOCCUS AUREUS INFECTION | -2,1205542 | 0 |
| GOBP_ENDOPLASMIC_RETICULUM_TO_GOLGI_VESICLE_MEDIATED_TRANSPORT | 2,1717203 | 0 | GOBP_PEPTIDE_ANTIGEN_ASSEMBLY_WITH_MHC_CLASS_II_PROTEIN_COMPLEX | -2,1202035 | 0 |
| GOBP_RESPONSE_TO_UNFOLDED_PROTEIN | 2,1558259 | 0 | GOBP_CELLULAR_EXTRAVASATION | -2,1160858 | 0 |
| GOBP_NEGATIVE_REGULATION_OF_MACROAUTOPHAGY | 2,154611 | 0 | GOBP_ANTIGEN_PROCESSING_AND_PRESENTATION_OF_EXOGENOUS_PEPTIDE_ANTIGEN_VIA_MHC_CLASS_II | -2,1025236 | 0 |
| GOBP_VESICLE_TARGETING | 2,1450226 | 0 | GOBP_ATTACHMENT_OF_SPINDLE_MICROTUBULES_TO_KINETOCHORE | -2,0972526 | 0 |
| GOBP_RESPONSE_TO_ENDOPLASMIC_RETICULUM_STRESS | 2,144913 | 0 | GOBP_ANTIGEN_PROCESSING_AND_PRESENTATION_OF_PEPTIDE_OR_POLYSACCHARIDE_ANTIGEN_VIA_MHC_CLASS_II | -2,0882614 | 0 |
| GOBP_MITOPHAGY | 2,1340785 | 0 | HEMATOPOIETIC CELL LINEAGE | -2,0853355 | 0 |
| GOBP_CELLULAR_RESPONSE_TO_UNFOLDED_PROTEIN | 2,1168478 | 0 | GOBP_KINETOCHORE_ORGANIZATION | -2,077481 | 0 |
| GOBP_AUTOPHAGIC_CELL_DEATH | 2,1128922 | 0 | GOBP_ANTIGEN_PROCESSING_AND_PRESENTATION_OF_EXOGENOUS_PEPTIDE_ANTIGEN | -2,0738037 | 0 |
| GOBP_PROCESS_UTILIZING_AUTOPHAGIC_MECHANISM | 2,0972846 | 0 | ASTHMA | -2,0718727 | 0 |
| GOBP_RESPONSE_TO_CADMIUM_ION | 2,0850573 | 0 | GOBP_STEROL_BIOSYNTHETIC_PROCESS | -2,0636125 | 0 |
| GOBP_REGULATION_OF_TRANSLATION_IN_RESPONSE_TO_STRESS | 2,0750623 | 0 | GOBP_NEUTROPHIL_MEDIATED_KILLING_OF_BACTERIUM | -2,0586567 | 0 |
| GOBP_REGULATION_OF_VACUOLE_ORGANIZATION | 2,0744436 | 0 | GOBP_CHONDROCYTE_PROLIFERATION | -2,0572882 | 0 |
| GOBP_REGULATION_OF_AUTOPHAGY | 2,0730388 | 0 | GOBP_MESENCHYMAL_CELL_PROLIFERATION | -2,0502887 | 0 |
| GOBP_RESPONSE_TO_MITOCHONDRIAL_DEPOLARISATION | 2,0589628 | 0 | GOBP_SPINDLE_MIDZONE_ASSEMBLY | -2,047648 | 0,0018657 |
| GOBP_PYRIMIDINE_CONTAINING_COMPOUND_TRANSMEMBRANE_TRANSPORT | 2,0539834 | 0 | GOBP_REGULATION_OF_MITOTIC_SISTER_CHROMATID_SEGREGATION | -2,0196402 | 0 |
| GOBP_VACUOLE_ORGANIZATION | 2,0495865 | 0 | GOBP_SPINDLE_CHECKPOINT_SIGNALING | -2,0183442 | 0 |
| GOBP_AUTOPHAGY_OF_MITOCHONDRION | 2,049519 | 0 | PROTEIN DIGESTION AND ABSORPTION | -2,0181768 | 0 |
| GOBP_VESICLE_TETHERING | 2,037076 | 0 | SYSTEMIC LUPUS ERYTHEMATOSUS | -1,99015 | 0 |
| GOBP_STRESS_GRANULE_ASSEMBLY | 2,021125 | 0 | GOBP_METANEPHROS_DEVELOPMENT | -1,9878899 | 0 |
| GOBP_REGULATION_OF_TRANSLATIONAL_INITIATION_BY_EIF2_ALPHA_PHOSPHORYLATION | 2,0185409 | 0 | GOBP_EPITHELIAL_CELL_CELL_ADHESION | -1,971351 | 0 |
| GOBP_NEGATIVE_REGULATION_OF_AUTOPHAGY | 2,017029 | 0 | GOBP_HETEROPHILIC_CELL_CELL_ADHESION_VIA_PLASMA_MEMBRANE_CELL_ADHESION_MOLECULES | -1,9679071 | 0 |
| GOBP_NUCLEOTIDE_PHOSPHORYLATION | 2,0091715 | 0 | GOBP_MEIOTIC_SPINDLE_ASSEMBLY | -1,951425 | 0 |
| GOBP_SELECTIVE_AUTOPHAGY | 2,0074985 | 0 | GOBP_COCHLEA_DEVELOPMENT | -1,9499881 | 0 |
| GOBP_MITOCHONDRIAL_ELECTRON_TRANSPORT_CYTOCHROME_C_TO_OXYGEN | 1,9994829 | 0 | GOBP_SPINDLE_ELONGATION | -1,9499338 | 0 |
| GOBP_ORGANELLE_DISASSEMBLY | 1,9968892 | 0 | GOBP_NEGATIVE_REGULATION_OF_CELL_JUNCTION_ASSEMBLY | -1,9497923 | 0,0018116 |
| GOBP_CELLULAR_RESPONSE_TO_LEUCINE_STARVATION | 1,9954473 | 0 | GOBP_POSITIVE_REGULATION_OF_MITOTIC_CELL_CYCLE_SPINDLE_ASSEMBLY_CHECKPOINT | -1,9361345 | 0,0018215 |
| GOBP_PROTEIN_DEMETHYLATION | 1,9807271 | 0 | BIOSYNTHESIS OF UNSATURATED FATTY ACIDS | -1,8193271 | 0,0016863 |
| GOBP_PYRUVATE_METABOLIC_PROCESS | 1,979806 | 0 | GRAFT-VERSUS-HOST DISEASE | -1,8112696 | 0 |
| GOBP_INTRA_GOLGI_VESICLE_MEDIATED_TRANSPORT | 1,9759649 | 0 | RHEUMATOID ARTHRITIS | -1,7703209 | 0,0034542 |
| GOBP_CELL_REDOX_HOMEOSTASIS | 1,9755392 | 0,0024331 | PPAR SIGNALING PATHWAY | -1,7530138 | 0,0033557 |
| GOBP_POSITIVE_REGULATION_OF_AUTOPHAGY | 1,9752611 | 0 | PROXIMAL TUBULE BICARBONATE RECLAMATION | -1,7382221 | 0,0110497 |
| GOBP_PEPTIDYL_PROLINE_HYDROXYLATION_TO_4_HYDROXY_L_PROLINE | 1,9731025 | 0 | STEROID BIOSYNTHESIS | -1,7123674 | 0,0159011 |
| GOBP_MITOCHONDRION_DISTRIBUTION | 1,9693602 | 0,0021739 | DNA REPLICATION | -1,6979182 | 0,0107335 |
| GOBP_PURINE_NUCLEOSIDE_DIPHOSPHATE_METABOLIC_PROCESS | 1,9681457 | 0 | LEISHMANIASIS | -1,6975935 | 0,003367 |
| GOBP_REGULATION_OF_AUTOPHAGY_OF_MITOCHONDRION_IN_RESPONSE_TO_MITOCHONDRIAL_DEPOLARIZATION | 1,9659002 | 0 | ANTIGEN PROCESSING AND PRESENTATION | -1,6836245 | 0 |
| GOBP_AUTOPHAGOSOME_ORGANIZATION | 1,9624032 | 0 | GLYCINE, SERINE AND THREONINE METABOLISM | 1,6993119 | 0 |
| GOBP_NEGATIVE_REGULATION_OF_TOR_SIGNALING | 1,9601648 | 0 | GLUTATHIONE METABOLISM | 1,7115604 | 0,0056022 |
| GOBP_REGULATION_OF_TRANSLATION_IN_RESPONSE_TO_ENDOPLASMIC_RETICULUM_STRESS | 1,9561042 | 0 | PANCREATIC CANCER | 1,7211257 | 0,0025575 |
| GOBP_POSITIVE_REGULATION_OF_MITOPHAGY_IN_RESPONSE_TO_MITOCHONDRIAL_DEPOLARIZATION | 1,9554675 | 0 | MTOR SIGNALING PATHWAY | 1,7475514 | 0 |
