# Supplementary material for: Metformin impacts the differentiation of mouse bone marrow cells into macrophages affecting tumour immunity
Source: Heliyon. 2024 Sep 11;10(18):e37792. doi: 10.1016/j.heliyon.2024.e37792 (PMC11417223; doi:10.1016/j.heliyon.2024.e37792)
Supplement: Multimedia component 4 [file mmc4.docx]

**Table S4. List of metabolites modified in metformin-treated BMDMs.**

| Metabolite | Mean AUC CTR | Mean AUC Met 2mM | Difference ± SEM | P-value |
| --- | --- | --- | --- | --- |
| Alanine/β-alanine | 113.9 | 106.7 | -7.232 ± 2.632 | 0.0515 |
| Arginine | 33.65 | 32.73 | -0.9244 ± 0.6976 | 0.2558 |
| Asparagine | 0.9754 | 0.9398 | -0.03556 ± 0.04836 | 0.5029 |
| Aspartic acid | 3.283 | 2.172 | -1.111 ± 0.04874 | <0.0001 |
| Glycine | 2.844 | 2.272 | -0.5723 ± 0.05972 | 0.0007 |
| Leucine | 18.44 | 17.64 | -0.8009 ± 0.5533 | 0.2213 |
| I-isoleucine | 6.708 | 6.212 | -0.4963 ± 0.1960 | 0.0645 |
| L-lactic acid | 50.74 | 135.90 | 85.16 ± 5.346 | <0.0001 |
| L-ornithine | 1.541 | 1.013 | -0.5272 ± 0.002793 | <0.0001 |
| Methionine | 3.925 | 3.721 | -0.2040 ± 0.1050 | 0.1238 |
| Phenylalanine | 9.290 | 8.310 | -0.9802 ± 0.2508 | 0.0174 |
| Proline | 10.44 | 7.313 | -3.129 ± 0.3025 | 0.0005 |
| Pyruvic acid | 1.4330 | 0.8146 | -0.6183 ± 0.1092 | 0.0048 |
| Serine | 9.819 | 9.791 | -0.02841 ± 0.2196 | 0.9033 |
| Valine | 11.07 | 10.76 | -0.3090 ± 0.3058 | 0.3694 |
| Glucose | 82.90 | 33.32 | -49.58 ± 0.9965 | <0.0001 |
| Citrulline | 1.835 | 1.789 | -0.04611 ± 0.05963 | 0.4825 |
| Malic acid | 18.65 | 21.60 | 2.945 ± 0.3938 | 0.0017 |
| Fumaric acid | 1.071 | 1.230 | 0.1587 ± 0.02555 | 0.0034 |
| Glutamic acid | 206.1 | 146.7 | -59.44 ± 3.686 | <0.0001 |
| Glutamine | 208.6 | 269.2 | 60.59 ± 6.867 | 0.0009 |
