# Supplementary material for: Metformin impacts the differentiation of mouse bone marrow cells into macrophages affecting tumour immunity
Source: Heliyon. 2024 Sep 11;10(18):e37792. doi: 10.1016/j.heliyon.2024.e37792 (PMC11417223; doi:10.1016/j.heliyon.2024.e37792)
Supplement: Multimedia component 5 [file mmc5.docx]

**Table S5. List of antibodies used for FACS immune-phenotyping and western blotting.**

| FACS immune-phenotyping | | | | |
| --- | --- | --- | --- | --- |
| Antibody | **Vendor** | **Cat No** | **µl/tube (100 µl)** | **RRID** |
| TruStain fcX | Biolegend | 101320 | 1µL | AB_1574975 |
| CD11b PerCP_Cy5.5 | Biolegend | 101227 | 5µL | AB_893233 |
| F4/80 PE | Biolegend | 123110 | 5µL | AB_893486 |
| CD80 BV510 | Biolegend | 104741 | 2.5µL | AB_2810337 |
| CD86 BV605 | Biolegend | 105037 | 5µL | AB_11204429 |
| H2Kb/H2Db PE/Cy7 | Biolegend | 114615 | 2.5µL | AB_2750195 |
| I-Ab APC | Biolegend | 116418 | 0.5µL | AB_10574160 |
| LIVE/DEAD™ Fixable Near-IR | Invitrogen | L34975 | 1µL | NA |
| Western blotting | | | | |
| Antibody | **Vendor** | **Cat No** | **Dilution** | **RRID** |
| Mouse anti-HIF1α | Abcam | Ab463 | 1:500 | AB_304539 |
| Rabbit anti-LC3 | Cell signaling | 2775S | 1:1000 | NA |
| Guinea pig anti-p62 | Progen | GP62-C | 1:1000 | AB_2687531 |
| Mouse anti-ACTIN | Millipore | MAB1501 | 1:10000 | AB_2223041 |
| Anti-mouse IgG, HRP-linked | Cell Signaling | 7076 | 1:20000 | NA |
| Anti-rabbit IgG, HRP-linked | Cell Signaling | 7074 | 1:20000 | NA |
| Anti-guinea pig IgG, HRP-linked | Jackson ImmunoResearch | 106-035-003 | 1:20000 | AB_2337402 |
