# Supplementary material for: Metformin impacts the differentiation of mouse bone marrow cells into macrophages affecting tumour immunity
Source: Heliyon. 2024 Sep 11;10(18):e37792. doi: 10.1016/j.heliyon.2024.e37792 (PMC11417223; doi:10.1016/j.heliyon.2024.e37792)
Supplement: Multimedia component 6 [file mmc6.docx]

**Table S6. Top 200 M1 & M2 signature genes (from Orecchioni et al. Front Immunol. 2019).**

| **M1** | | | | | **M2** | | | | |
| --- | --- | --- | --- | --- | --- | --- | --- | --- | --- |
| Wipi1 | Acyp2 | Aox1 | D530039A21Rik | Irf9 | Arg1 | Rrm2 | Zyx | Cenpf | Serpina3g |
| Mlx | Dnajc1 | Sash1 | Col4a2 | Cmklr1 | Mgl2 | Trem2 | Ppbp | Pros1 | Cttn |
| March1 | Psmb8 | Psmd10 | Mctp1 | Cd82 | Tmem26 | Mmp9 | Gpc1 | Deptor | Eps8 |
| Gdi1 | Mrpl45 | Ero1l | Slc41a1 | Pwp1 | Rnase2a | Zranb3 | Chst7 | Arhgap6 | Cpeb1 |
| Zdhhc6 | Rras | Acbd4 | Tmem119 | Slamf6 | Mrc1 | Ephx1 | Plk2 | Cass4 | Rnf144b |
| Stam | Tmem63a | Snx20 | Gtf2h2 | Abhd3 | Egr2 | Ccnb2 | Ch25h | Gnb4 | Nuf2 |
| Prag1 | Amn1 | P2ry13 | Adgre1 | Neu1 | Flt1 | Cd300ld | Cd83 | Kif20a | Etv5 |
| Coro1a | Slx4ip | Ppm1n | Rffl | Snx10 | Chil3 | Rad51 | Ryr1 | Mcm7 | Enpp1 |
| Gpcpd1 | Xylt2 | Gabpb1 | Trafd1 |  | Clec10a | Irf4 | Rnase6 | Fam132a |  |
| Usp3 | Pten | Rhov | Ralgps1 |  | Atp6v0d2 | Emp2 | Cdca3 | Cnrip1 |  |
| Ccdc88b | Dnajb4 | Igsf6 | Fkbpl |  | Fam198b | Efr3b | Car2 | Shcbp1 |  |
| Taf1b | Msr1 | Nmi | Zcchc4 |  | Matk | Klf9 | Cd28 | F2r |  |
| Rdh11 | Ifi27l2a | A430093F15Rik | Tdrd7 |  | Socs2 | P2ry1 | Mxd4 | E2f7 |  |
| Mefv | Cwc27 | Ntpcr | Plekhn1 |  | Itgb3 | Ets1 | Fyn | Gcnt1 |  |
| Faap20 | Kcna3 | AU015263 | P4ha1 |  | Ocstamp | Ccna2 | Uhrf1 | Ptpro |  |
| Fcho1 | Exoc3l4 | Slc2a6 | Slc39a4 |  | Ptgs1 | Egr3 | Mcm5 | Slamf1 |  |
| Nab1 | Fmnl3 | Slc16a3 | Smox |  | S100a4 | Tmem267 | Asap2 | Racgap1 |  |
| Cmtr1 | Ly6i | Mvd | Vamp2 |  | Clec7a | Clec4b1 | Dut | Gpr183 |  |
| Gstt4 | Slc15a3 | E030030I06Rik | Hspa4l |  | Plxdc2 | Birc5 | H2-Eb1 | Dnmt3a |  |
| 6720422M22Rik | Rassf4 | Pik3cg | Lrrc4 |  | Hbegf | Cbr2 | Il7r | Hebp2 |  |
| Tnfrsf21 | Adhfe1 | 9630050P21Rik | Kbtbd7 |  | Ccl24 | Fn1 | Tmem176b | Alms1 |  |
| Nfxl1 | Kctd1 | BC017643 | Marf1 |  | Emp1 | Edn1 | Tacc2 | Mafb |  |
| Pim2 | Slc16a6 | Sntb2 | Syk |  | Pdcd1lg2 | Stmn1 | H2-Aa | Tfrc |  |
| Map3k14 | Htra2 | Zfas1 | Mtdh |  | Olfm1 | Igf1 | Socs6 | Cd74 |  |
| Cxcl16 | Def8 | Ophn1 | Slc25a19 |  | Ube2c | Pkp2 | Hfe | Slc1a5 |  |
| Kif13b | 6330407A03Rik | Slc8b1 | Disp1 |  | Vwf | Tmem176a | Ckb | Prr15 |  |
| Slc6a13 | Fdft1 | Trps1 | Fam134b |  | Crip1 | Hmga2 | Angptl2 | Gpt2 |  |
| Scamp1 | Axl | St3gal1 | Morc3 |  | Pparg | Myc | Ahr | Ank |  |
| Tpd52 | Pla2g7 | Mdm4 | Armc8 |  | Btbd11 | Atp6v0a1 | Bhlhe40 | Dok2 |  |
| Ssh1 | Cdc42bpg | Ctu1 | Rasgrp1 |  | Flrt2 | Il1rl1 | Ccnd1 | Kif11 |  |
| Snapc1 | Prorsd1 | Abcc4 | Ncoa7 |  | Rab3il1 | Sfpq | Stab2 | Ffar4 |  |
| Ift22 | Whamm | Ralgps2 | Slc20a1 |  | St6gal1 | Hip1 | Pbk | Psat1 |  |
| Epha1 | Slc1a2 | Usb1 | Vps37a |  | Mmp12 | Rad51ap1 | C79468 | Arap2 |  |
| Stra6l | Ptch1 | Sgk1 | Lgals3bp |  | Dcstamp | Tmem158 | Chn2 | Rgs16 |  |
| Ppp1r11 | Zbtb7b | Slfn2 | S1pr1 |  | Ccl17 | Ednrb | Mtss1 | Slc25a13 |  |
| Sh3bp5 | Bmp1 | Trim25 | Atxn7l1 |  | Tiam1 | Dab2 | Hacd1 | C86753 |  |
| Crcp | H2-K1 | Pirb | Rnf114 |  | Rhoj | Tfec | Zfp36l1 | AI956758 |  |
| Dnajc18 | Aif1 | Dram1 | Traf2 |  | Rras2 | Lmna | Tle1 | Ppp1r1a |  |
| Ccdc50 | Gmfg | Sirt3 | Pfkl |  | Pclaf | Tanc2 | S100a6 | Cenpa |  |
| Camp | Alkbh2 | Ginm1 | Grk2 |  | Il6st | Daglb | Nrp1 | Dusp4 |  |
| Slc25a12 | Apbb3 | Mocs1 | Raf1 |  | Fcrls | Tbc1d4 | Rgs2 | Clstn1 |  |
| Chst1 | Nxpe3 | Kif3c | Arl6 |  | Itgax | Amz1 | Slc39a10 | Man1a |  |
| Dnm1l | Slc6a12 | Batf2 | Tmem134 |  | Bcar3 | Fabp4 | D11Ertd717e | Nav1 |  |
| Ttc39b | 1810043G02Rik | Epsti1 | Cdc42ep2 |  | Plekhf1 | Fgf13 | Gpr68 | Car5b |  |
| Sirpb1a | Grap | Tor1a | Ptk2b |  | Batf3 | Slc9a9 | Tcf19 | Wtip |  |
| Lhfpl2 | Vcam1 | Slc2a3 | Mov10 |  | Cd36 | Cish | Anln | Pcdh7 |  |
| Ifitm6 | Ccnd2 | Rsph9 | Samhd1 |  | Cd300lb | Cdc20 | Kif2c | Lrmp |  |
| Pnkd | Hspb6 | 9530006C21Rik | Stat3 |  | Timp2 | Tox2 | Lat | Rgs18 |  |
