# Supplementary material for: Metformin impacts the differentiation of mouse bone marrow cells into macrophages affecting tumour immunity
Source: Heliyon. 2024 Sep 11;10(18):e37792. doi: 10.1016/j.heliyon.2024.e37792 (PMC11417223; doi:10.1016/j.heliyon.2024.e37792)
Supplement: Multimedia component 7 [file mmc7.docx]

**Table S7. List of primers used for qPCR analyses.**

| **Gene name** | **Supplier** | **Reference** | **Forward primer sequence (3’-5’)** | **Reverse primer sequence (3’-5’)** |
| --- | --- | --- | --- | --- |
| *Rpl27* | Eurogentec | NA | TGGAATTGACCGCTATCCCC | CCTGTCTTGTATCGCTCCTCAA |
| *Tgfbi* | Eurogentec | NA | GCCCCAATGTATGTGCTGTG | ATTTGAGACCGGAAGAGCTGC |
| *Il1b* | Eurogentec | NA | AGCTTCCTTGTGCAAGTGTCT | GACAGCCCAGGTCAAAGGTT |
| *Ccl2* | Eurogentec | NA | CACTCACCTGCTGCTACTCATC | GCTTCTTTGGGACACCTGCTG |
| *Tnf* | Eurogentec | NA | GGTTCTGTCCCTTTCACTCAC | TGCCTCTTCTGCCAGTTCC |
| *Vegfa* | Eurogentec | NA | CTCCACCATGCCAAGTGGTC | CACTCCAGGGCTTCATCGTT |
| *Il6* | Eurogentec | NA | ACCGCTATGAAGTTCCTCTC | CTCTGTGAAGTCTCCTCTCC |
| *Tgfb1* | Eurogentec | NA | GCCACAAACGGTGTGGTCTA | GCTGATAGACAGGGGCAAGTC |
| *Arg1* | Eurogentec | NA | AGACAGCAGAGGAGGTGAAGAG | CGAAGCAAGCCAAGGTTAAAGC |
| *Il10* | Eurogentec | NA | GGCGCTGTCATCGATTTCTC | ATGGCGTTGTAGACACCTTGG |
| *H2-Aa* | Eurogentec | NA | TCTGTGGAGGTGAAGACGAC | AGGAGCCTCATTGGTAGCTGG |
| *Ly6c1* | Eurogentec | NA | GTGTGCAGAAAGAGCTCAGG | GAAAGGCACTGACGGGTCTT |
| *Cd74* | Eurogentec | NA | GACCCAGGACCATGTGATGC | TTCCTGGCACTTGGTCAGTACTTTA |
| *Mthfd2* | Eurogentec | NA | CTGCGTTGGCTGTGCGGTT | CTGAGGTGTGGCCGCTTGT |
| *Car9* | Qiagen | PPM03340A-200 | NA | NA |
