# Supplementary material for: Metformin impacts the differentiation of mouse bone marrow cells into macrophages affecting tumour immunity
Source: Heliyon. 2024 Sep 11;10(18):e37792. doi: 10.1016/j.heliyon.2024.e37792 (PMC11417223; doi:10.1016/j.heliyon.2024.e37792)
Supplement: Multimedia component 8 [file mmc8.docx]

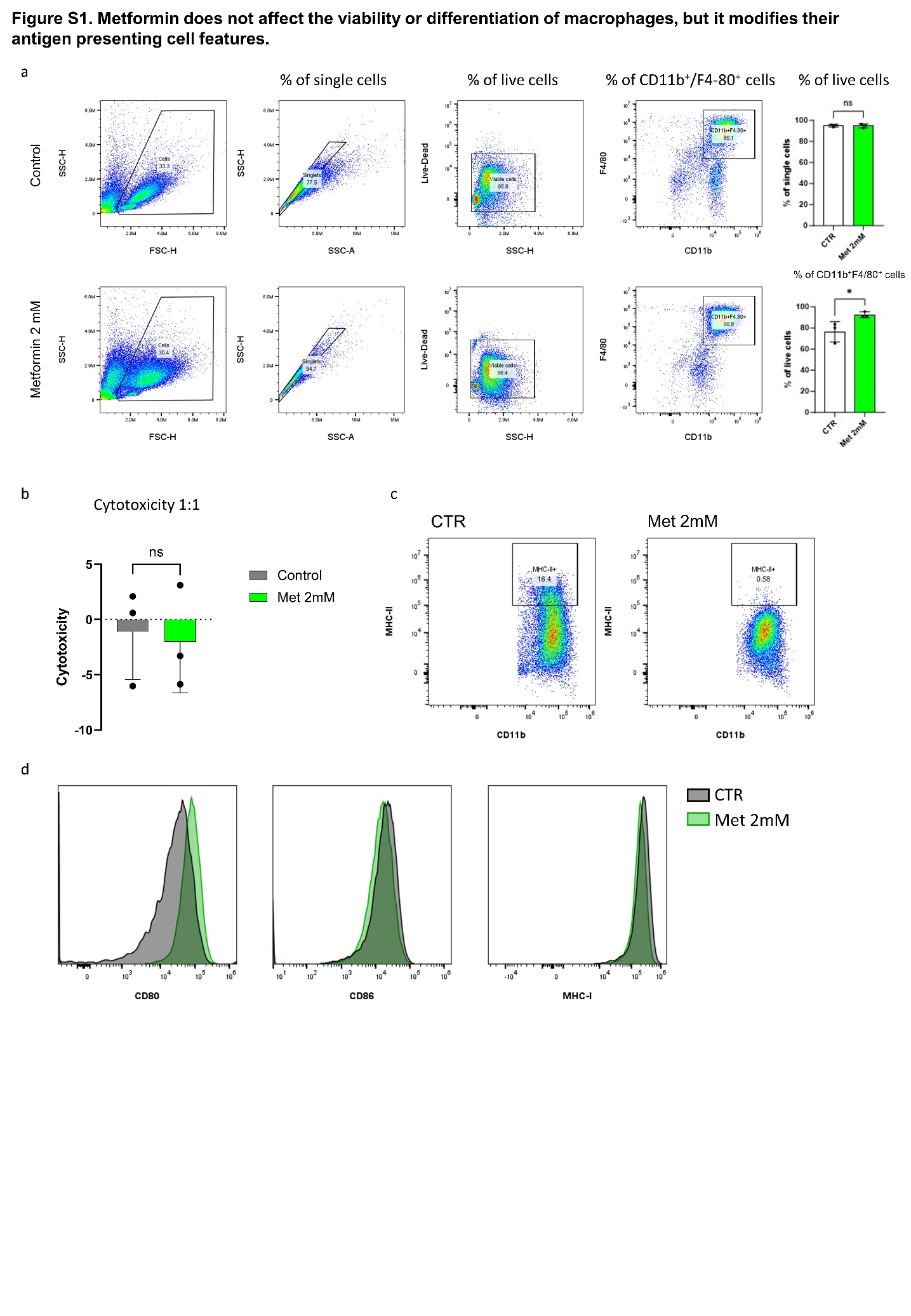


## Supplementary figure 1. Metformin does not affect the viability or differentiation of macrophages, but it modifies their antigen presenting cell features.

**(A)** Gating strategy of bone marrow-derived macrophages untreated (up) and treated (down) with metformin (2 mM) to quantify the amount of live cells and the percentage of fully differentiated macrophages based on the positivity for CD11b and F4/80 by flow cytometry. Unpaired t-test. Graphs show percentage ± SD. Ns=p>0.05 (n = 3 biological replicates). **(B)** Percentage of dead GL261 cells after co-culture with treated or untreated bone marrow-derived macrophages in an effector:target ratio 1:1. Graph shows percentage ± SD. Paired t-test. Ns=p>0.05 (n = 3 biological replicates). **(C)** Gating strategy used to quantify the percentages of MHC class II^+^ cells among CD11b^+^ cells between metformin-treated and untreated conditions. **(D)** Representative gating histograms of CD80, CD86 and MHC-I expression levels of untreated (grey) and metformin-treated (green) BMDMs.


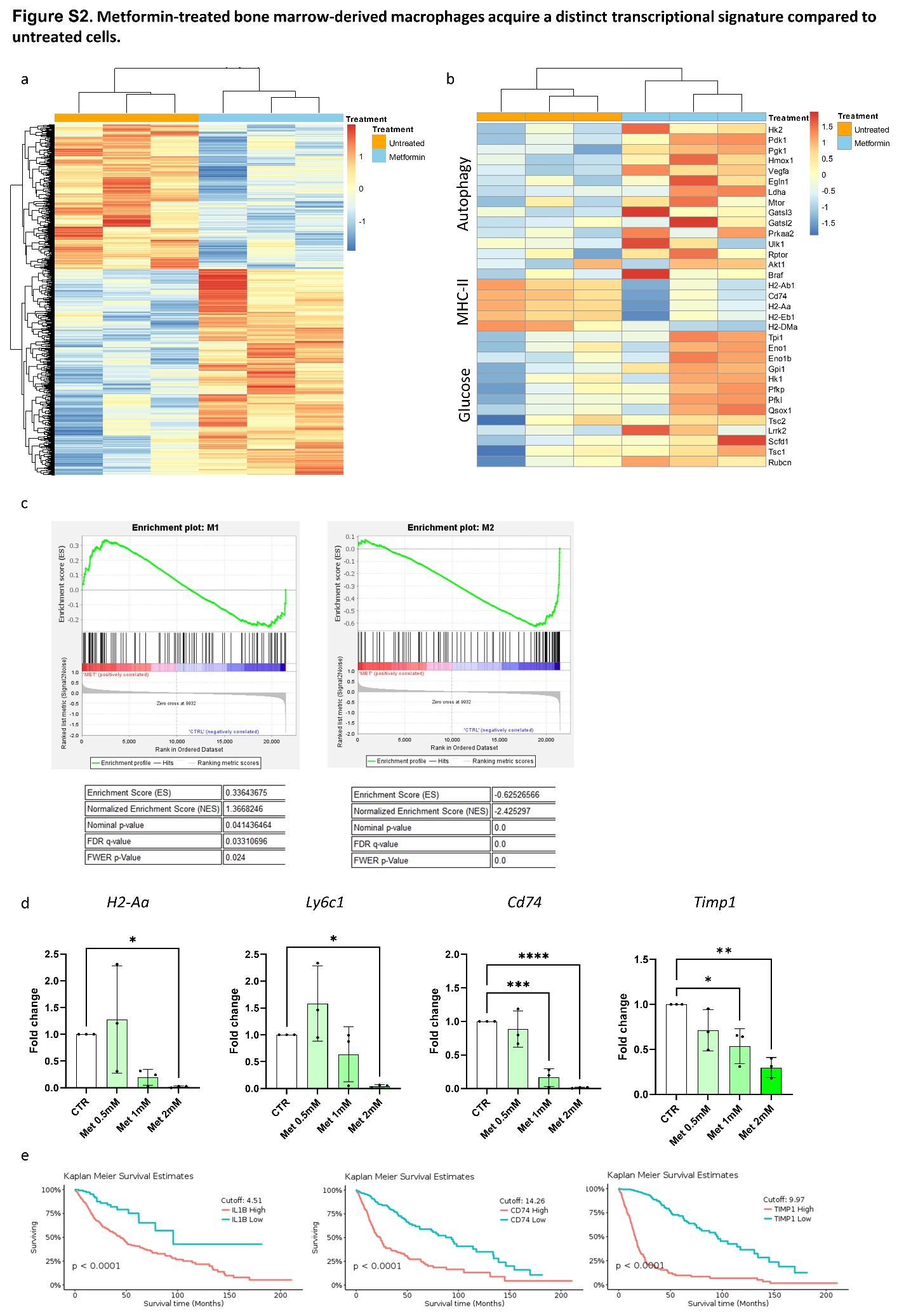


## Supplementary figure 2. Metformin-treated bone marrow-derived macrophages acquire a distinct transcriptional signature compared to untreated cells.

**(A)** Heatmap showing significantly regulated genes between metformin-treated and untreated bone marrow-derived macrophages (p-value < 0.05, |log2FC| ≥ 0.5). Color bar shows z-scores of expression. **(B)** Heatmap showing expression of preselected genes involved in regulation of autophagy (autophagy), antigen presentation via MHC class II (MHC-II), and glucose catabolic process (glucose), modulated between metformin-treated and untreated bone marrow-derived macrophages. Color bar shows z-scores of expression. **(C)** Relative quantification of the expression of *H2Aa*, *Ly6c1*, *Cd74* and *Timp1* in macrophages that have been treated with 3 different concentrations of metformin. Graphs show fold change compared to untreated BMDMs ± SD. One-way ANOVA. *=p<0.05, ***=p<0.001, ****=p<0.0005 (n = 3 biological replicates). **(D)** Kaplan Meier curves showing survival time of glioma patients stratified based on *IL1B*, *CD74* and *TIMP1* mRNA expression in the tumour tissue. **(E)** Gene set enrichment analysis plots comparing the transcriptional signature of metformin-treated macrophages with M1 and M2 prototypical signatures.
